# Scenario planning with linked land-sea models inform where forest conservation actions will promote coral reef resilience

**DOI:** 10.1038/s41598-018-29951-0

**Published:** 2018-08-20

**Authors:** J. M. S. Delevaux, S. D. Jupiter, K. A. Stamoulis, L. L. Bremer, A. S. Wenger, R. Dacks, P. Garrod, K. A. Falinski, T. Ticktin

**Affiliations:** 1 Department of Botany, University of Hawaiʻi, Honolulu, HI USA; 2 School of Ocean and Earth Science and Technology, University of Hawaiʻi, Honolulu, HI USA; 3 Wildlife Conservation Society, Melanesia Program, 11 Ma’afu Street, Suva, Fiji; 4 School of Molecular and Life Sciences, Curtin University, Perth, Australia; 5 Fisheries Ecology Research Lab, University of Hawaiʻi, Honolulu, HI USA; 6 University of Hawaiʻi Economic Research Organization, University of Hawaiʻi, Honolulu, HI USA; 7 University of Hawaiʻi Water Resources Research Center, University of Hawaiʻi, Honolulu, HI USA; 8 School of Earth and Environmental Sciences, University of Queensland, Brisbane, QLD Australia; 9 Department of Biology, University of Hawaiʻi, Honolulu, HI USA; 10 Department of Natural Resources and Environmental Management, University of Hawaiʻi, Honolulu, HI USA; 11 The Nature Conservancy, Hawaiʻi Marine Program, Honolulu, HI USA

## Abstract

We developed a linked land-sea modeling framework based on remote sensing and empirical data, which couples sediment export and coral reef models at fine spatial resolution. This spatially-explicit (60 × 60 m) framework simultaneously tracks changes in multiple benthic and fish indicators as a function of land-use and climate change scenarios. We applied this framework in Kubulau District, Fiji, to investigate the effects of logging, agriculture expansion, and restoration on coral reef resilience. Under the deforestation scenario, models projected a 4.5-fold sediment increase (>7,000 t. yr^−1^) coupled with a significant decrease in benthic habitat quality across 1,940 ha and a reef fish biomass loss of 60.6 t. Under the restoration scenario, models projected a small (<30 t. yr^−1^) decrease in exported sediments, resulting in a significant increase in benthic habitat quality across 577 ha and a fish biomass gain of 5.7 t. The decrease in benthic habitat quality and loss of fish biomass were greater when combining climate change and deforestation scenarios. We evaluated where land-use change and bleaching scenarios would impact sediment runoff and downstream coral reefs to identify priority areas on land, where conservation or restoration could promote coral reef resilience in the face of climate change.

## Introduction

Over the past half century, climate change has increasingly impacted global coral reefs through bleaching^1^, ocean acidification^2^, and intensified storms^3^. Concurrently, anthropogenic land-use change at a local scale has altered terrestrial fluxes of freshwater^4^, sediments^5^, and nutrients^6,7^ to coral reef environments, thereby threatening over 25% of the total global reef area^8^. Across much of the tropics, logging and commercial agriculture expansion, in particular, are threatening coral reefs through increased sediment and nutrient runoff^9,10^. Excess nutrients and sediments have been shown to impact coral reefs by promoting benthic algae growth and smothering corals, respectively^11–13^. Nutrients are known to bind to and travel with sediment, thereby potentially contributing to lack of recovery from bleaching through promoting algae growth^14,15^. In some cases, high turbidity and suspended sediment have been shown to mitigate elevated sea surface temperature (SST) and associated bleaching impacts^16,17^. Although the extent to which sediment and nutrient levels interact with elevated SST to affect the outcome of bleaching events remains poorly understood, it is increasingly recognized that water quality plays a complex role in the fate of nearshore coral reefs under climate change^16–18^.

Today’s challenge for conservation science and resource management is understanding how to manage co-occurring global and local human impacts to foster coral reef resilience^19,20^. These trends have led to the decline of important resources upon which human wellbeing depends^21,22^. Thus, managing the impacts of local human drivers has been widely advocated to promote resilience of coral reefs in the face of climate change^23^, though the effectiveness of these efforts can differ among places^24,25^. Anthropogenic impacts vary in space and time depending on land-use change intensity and extent, topography and bathymetry (e.g., landscape steepness and reef slopes), and geology (e.g., soil type, benthic habitat). As a result, efforts to address coral reef degradation through sustainable land management will have differential impacts on coral reefs based these underlying differences^26^. Historically, terrestrial and marine ecosystems have been managed disparately, where terrestrial protected areas (TPAs) and marine protected areas (MPAs) are often designed and allocated regardless of downstream or upstream activities^27,28^. MPAs are not effective at addressing land-based source pollution impacts on coral reefs^29^, while TPAs can foster downstream benefits when accounting for land-sea linkages^30–32^. Accounting for these linkages when placing TPAs could help increase benefits and resilience of both terrestrial and marine ecosystems under a changing climate^33^. Therefore, decisions about where to protect coral reefs and forest ecosystems need to be supported by spatial conservation prioritization analyses and tools to understand the potential cumulative impacts and multiple outcomes of these decisions^32,34^.

Managing the impact of terrestrial runoff on coral reefs requires tracing its sources and identifying potential impacts downstream. However, identifying pollutant sources is challenging because of multi-scale processes affecting pollutant export, retention, and eventual impacts on coral reefs. For instance, deforestation can occur in multiple watersheds and increase sediment runoff into the coastal zone^35–37^. Once in the ocean, sediment is dispersed by nearshore processes and impacts coral reefs through decreased water clarity, shading, or smothering of benthic organisms, thereby resulting in reduced habitat quality and direct and indirect negative impacts to some reef fish taxa^38–40^. The location and extent of coral reef impacts from sediment runoff also depend on marine drivers such as waves, tides, and nearshore transport^41^. These numerous processes operating at multiple spatial and temporal scales create real challenges for identifying specific geographies for terrestrial actions that will mitigate downstream impacts on the most affected reef areas^32,42^.

Around the Pacific, there is growing concern over the effects of logging and agriculture expansion on coral reef fisheries due to increased sediment runoff^9^, projected to be exacerbated under climate change impacts^43^. Consequently, communities seek to revitalize customary ridge-to-reef resource management systems to foster coral reef resilience in the face of climate change and declining resources, such as the revival of the *ahupua‘a* system in Hawaiʻi^44,45^ and integrated land-sea management that includes the concept of *vanua* in Fiji^46^. Integrated land-sea planning requires tools to understand the potential outcomes of management actions to inform spatial prioritization^32,34^. Complex models can capture these processes, yet these approaches are data intensive and can be costly to implement^34,47^. Many developing countries cannot afford the time and resources necessary to adopt these methods. For instance, communities and government in Fiji are developing provincial-level Integrated Coastal Management (ICM) plans to balance terrestrial and marine economies, as well as maintain ecological integrity, and therefore, require information on where forest conservation can reduce impacts to coral reefs^42^. However, limited funding and capacity hinders the development and implementation of decision support tools that can support these recent government ICM initiatives. Therefore, developing linked land-sea decision support tools that are flexible, simple to implement and interpret^48,49^, and rely on existing and freely available data and software are needed^42,48–50^.

Integrated land-sea planning requires decision-support tools that simultaneously evaluate the impacts of terrestrial and marine drivers on coral reefs at a fine spatial scale to be relevant for management^32,34,42^. In spite of existing conceptual frameworks to adopt land-sea planning^27,51^, only a few practical examples demonstrate how to operationalize ridge-to-reef concepts into conservation planning. Some applications have linked the effects of land-uses to marine ecosystems at broad spatial resolutions to identify regions for more refined analysis^26,33,52,53^. Some quantified the linkages between sediment loads and seagrass suitable habitat^54^. Others have demonstrated that incorporating spatially-explicit land-sea connections changes and improves prioritization of watersheds or coral reef areas for protected area establishment at finer spatial resolution (1 km^2^)^32,41,55,56^. However, these applications remain too coarse spatially and ecologically to prioritize areas for conservation at the sub-watershed scale and to inform ridge-to-reef management at a scale relevant to Pacific Islanders.

To address this gap, we leveraged a novel linked land-sea modeling framework, which was developed to inform sustainable development in Hawaiʻi at the sub-watershed scale^57^. This spatially-explicit (60 × 60 m) framework simultaneously tracks changes in multiple benthic and fish indicators under land-use and climate change scenarios. This framework offers a flexible approach because the component models can be adapted based on existing data and updated as new data become available. Another relevant approach is the open source Integrated Valuation of Ecosystem Services and Tradeoffs (InVEST) toolbox from the Natural Capital Project^49,58^. We leveraged the spatially-explicit Sediment Delivery Ratio Model (SDR version 3.2), which uses soil erosion equations to identify the land areas supplying sediment loads to stream mouths^59,60^. Combining the strengths of these two approaches holds promise for tropical data-poor regions. By coupling the SDR model and coral reef models from these two approaches, this study fills an important gap by providing spatial predictions of multiple benthic and reef fish indicators at high resolution in order to evaluate the effects of land-use and climate change on coral reef resilience.

We applied our spatially-explicit modeling framework with scenario planning in Kubulau (Fiji), where logging and commercial agriculture expansion competes with forest conservation and potentially fisheries livelihoods, like many ridge-to-reef systems across the Pacific^9^. We designed scenarios that represent extreme projected land-use change based on a land-use classification system developed by the Fiji Department of Agriculture (e.g., logging concessions, agriculture, and conservation classes) and commercial agriculture practices (e.g., polyculture and monoculture practices). To support the ongoing ICM efforts in Fiji and provide insight for linked watershed and reef systems on tropical high islands, we addressed the following research questions: (1) How does sediment runoff influence coral reef benthic and fish indicators? (2) Where do deforestation or restoration impact coral reefs due to differences in sediment runoff, while accounting for climate change? (3) Where are the most effective areas on land to prioritize forest conservation (i.e., prevent deforestation) and restoration to foster coral reef resilience? Our approach relies on existing data, including topography and bathymetry, land cover/use, and rainfall, which are becoming increasingly freely available, combined with locally sourced coral reef data. Thereby making this approach broadly applicable and transferable to data-limited regions, in order to support land-sea integrated planning.

## Methods

### Case study: Kubulau District, Fiji

**Figure 1:**
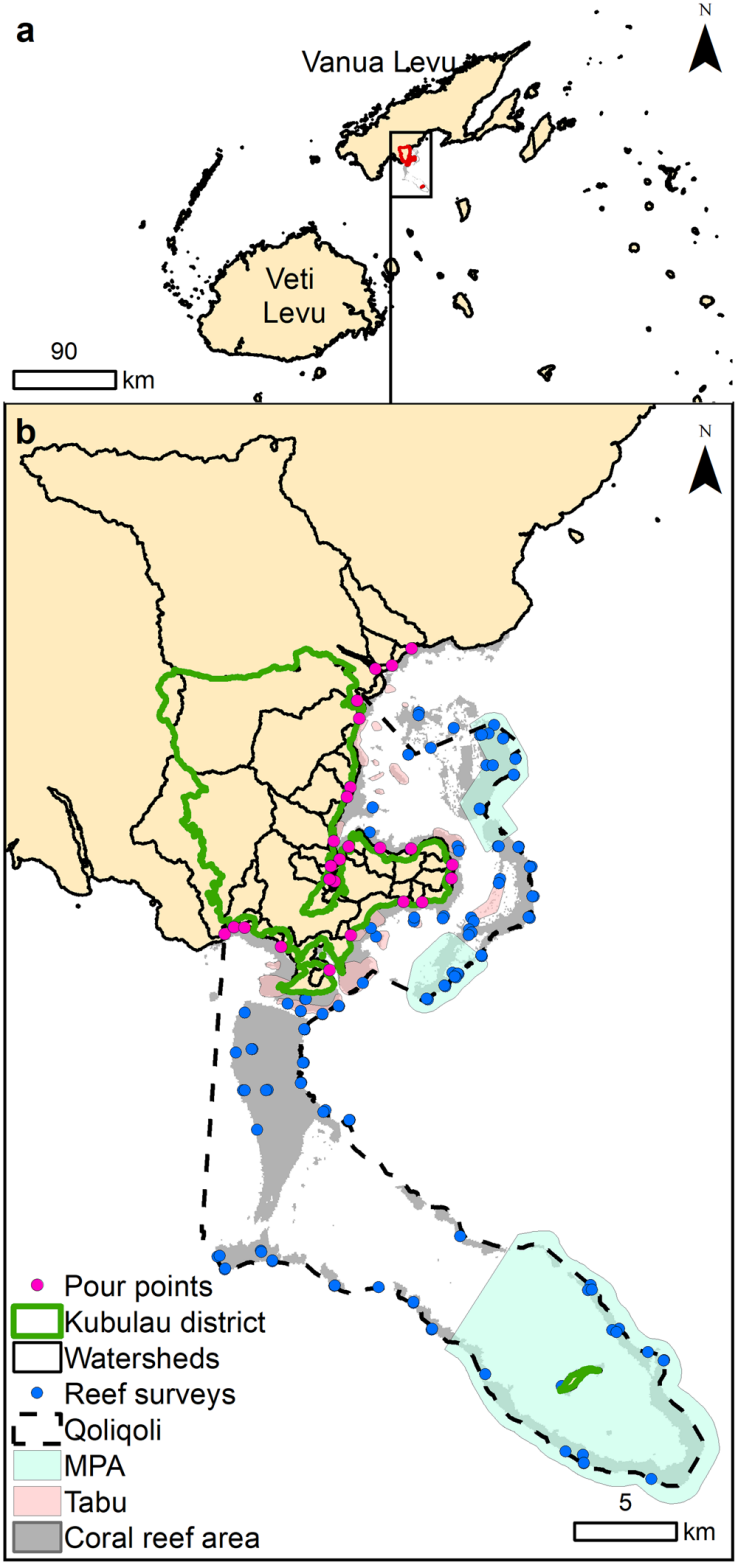
Study site. (**a**) Location of Kubulau District study site in Fiji. (**b**) Watershed boundaries, pour points, coral reef areas, and reef surveys are shown along with the customary fishing grounds (*qoliqoli*), periodically-harvested fisheries closures (*tabu*) and district level no-take MPAs.

Villages and governments on Vanua Levu, the second largest island in the Fijian Archipelago, are becoming increasingly concerned that economic development (e.g., commercial agriculture, forest logging expansion) is impacting fisheries, tourism, and the ecological integrity of coral reefs and nearshore ecosystems^61^. For instance, a 20-year logging concession agreement was signed in 2006 for the Bua province on Vanua Levu, where Kubulau is located (Fig. 1). Growing demand for wood and root crops (i.e., taro and kava) for export increases pressure to convert native forest to pine forest in logging concessions, while commercial agriculture expands on land and slopes suitable for taro and kava monoculture. To this day, Kubulau District still has 70–80% of its native forest cover with relatively intact hydrologic connectivity between terrestrial, freshwater, and marine areas^52,62^, except for introduced pine tree plantations from previous logging activities along the coast^52,62^.

To address these concerns, communities are currently working with government, NGOs and the private sector to design and implement ICM plans based on a national ICM frameworks for Fiji^63^. To address the tradeoffs inherent in designing ICM management interventions, information on priority areas for forest conservation or restoration that can promote coral reef ecosystem resilience under projected climate change is needed^63^. To support these efforts, we estimated the impact of projected land-use change on coral reefs under climate change scenarios in Kubulau District (population ~1,000) based on current and potential future logged areas. Our sediment model domain spanned all the watersheds discharging in traditional fishing grounds (*qoliqoli*), which include watersheds in Kubulau District and three large adjacent watersheds (Fig. 1b). In the customary fisheries management area (*qoliqoli*), coral reef habitats include fringing reefs, lagoons, patch reefs, and barrier reef extending over 30 km offshore. The local communities have implemented an MPA network of 21 community-managed periodically harvested fisheries closures (*tabu* areas) and three district-wide permanent no-take MPAs^64^.

### Modeling approach overview

**Figure 2:**
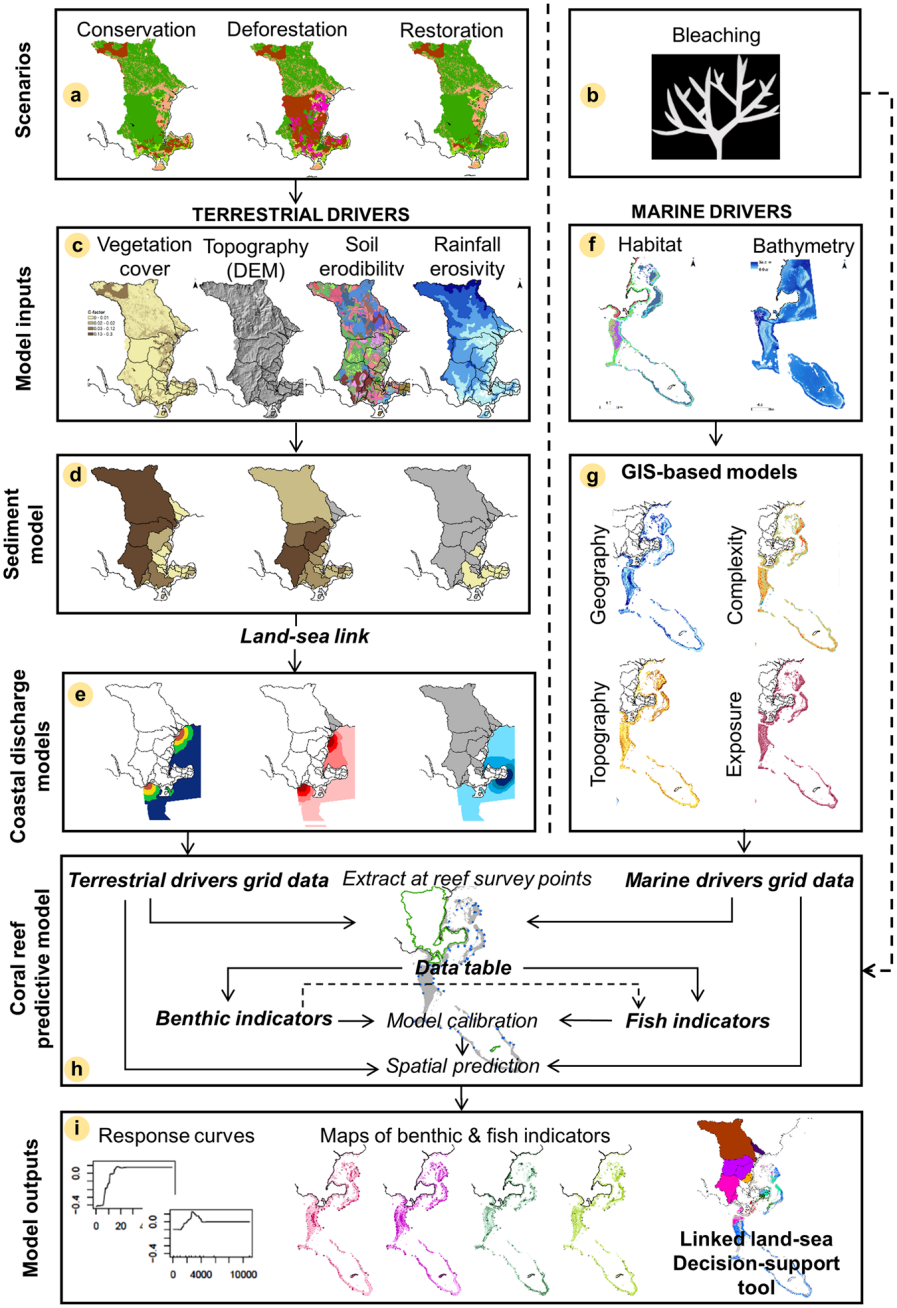
Linked land-sea modeling framework. (**a**,**b**) Land-use and climate change scenarios were coupled with the linked land-sea framework^163^. (**c**) Land cover, topography, soil, and climate data were inputs in (**d**) the InVEST Sediment Delivery Ratio (SDR) model to quantify sediment export (t.yr^−1^). (**e**) The coastal discharge models used GIS distance-based dispersion models to generate the terrestrial driver grid data (t.yr^−1^). (**f**) Bathymetry and habitat maps were combined with (**g**) GIS-based models to derive the marine driver grid data (i.e., habitat topography, geography, exposure and complexity). (**h**) The coral reef predictive models were calibrated on coral reef survey data. (**i**) Outputs were: (1) response curves, (2) maps of benthic (% cover) and fish (kg.ha^−1^) indicators, and (3) a linked land-sea decision-support tool.

To spatially prioritize terrestrial conservation efforts across the landscape based on downstream coral reef impacts, we determined the impacts of projected climate change and land-use change on coral reefs and traced those back to the areas driving these impacts within each watershed. In order to do so, we coupled various land-use scenarios with an adapted linked land-sea modeling framework^57^ (Fig. 2). Previously developed for quantifying the effect of nutrient enriched groundwater on coral reefs, the modified framework links InVEST SDR version 3.2^59,60^ to coral reef ecosystem state models calibrated with existing empirical and remote sensing data. Although nutrients are associated with sediment runoff and agriculture expansion^11^, we were not able to explicitly model nutrient runoff here because of the lack of spatially-explicit information on local fertilizer application rates. First, we designed land-use change scenarios that represent extreme projections of where deforestation and restoration could occur using the national land-use classification system and agriculture practices, and climate change scenarios based on historical bleaching impact in Fiji and models developed for similar latitudes in Hawaiʻi. Second, we adapted, calibrated and applied the land-sea framework, which connects the sediment and coral reef models. To measure proxies of ecological resilience, the coral reef models focused on four benthic indicators, known to respond to land-based runoff, and four fish indicators, which represent important subsistence and cultural resources^13,62,65^. Last, we undertook a spatial analysis to assess the impact of future scenarios on coral reefs and identify priority areas in specific watersheds, where avoiding deforestation through conservation or promoting restoration could foster coral reef resilience in the face of climate change.

### Human drivers scenarios

#### Land-use change scenarios

We considered three feasible land-use change scenarios (maintenance of current land-use, deforestation, and restoration) that represent alternative projected and extreme land-use change in Kubulau and generally in many other watersheds across tropical high islands^9^. The scenarios were designed based on a land-use capability (LUC) classification map developed by Fiji Department of Agriculture (Land Use Section) to inform their spatial land-use planning^66^. This LUC classification system is based on land development planning, soil conservation, and promotion of sustainable land-use practices. The LUC classification has eight classes: classes I–IV include land suitable for arable cultivation; classes V–VII include land suitable only for pastoral or forestry use; and class VIII is land suitable only for conservation. Each LUC class includes a range of natural limitations with respect to human uses after accounting for the physical qualities of the soil and the surrounding environment. Limitations were assessed by the Department of Agriculture based on susceptibility to erosion, steepness of slope, susceptibility to flooding, wetness, drought, salinity, depth of soil, soil texture, stoniness, structure, nutrient supply and climate, which in turn affect productivity, land-use practices, and type of land-use and associated intensity^66^.

In ArcGIS, we reclassified the current land-use of each pixel to future land-use based on the LUC class. Under the maintenance of the current land-use scenario, all the existing native forest is placed under conservation. The current land cover was derived from satellite imagery (see Brown *et al.*^42^ for more details). Under the deforestation scenario, we assumed that all the areas suitable for logging (LUC: V–VII) within designated current and proposed logging areas (logging concessions) were cleared and converted to plantations of pine (*Pinus caribaea*), an introduced timber and the principal forestry species in Fiji (described in Jupiter *et al*.^67^). Outside of logging concession areas, areas suitable for agriculture (LUC I–IV) were converted to commercial agriculture, such as monoculture of taro (*Colocasia esculenta*) and kava (*Piper methysticum*). These two root crops are the most important cash crops in this part of Vanua Levu and important drivers of deforestation across many Pacific Islands^68^. We assumed that existing native forest located in LUC VIII were not deforested. Under the forest restoration scenario, we assumed no loss of the current cover of native forest and all the existing pine plantations from previous logging activities were converted back to native forest, a strategy promoted by government and NGOs through payment for ecosystem services (PES) schemes to reduce soil erosion^69^. While most efforts include restoration with introduced species^70^, we assume restoration to native forest because of its conservation value^71^.

#### Climate change scenarios

Three climate change scenarios (low, moderate, and high impact in terms of levels of coral bleaching) were designed to assess the potential impact of increases in SST and atmospheric carbon dioxide (CO_2_) on coral reefs. The coral bleaching scenarios were derived from recorded and projected coral bleaching impacts for the region^72,73^. An average greenhouse gas emissions scenario (A1) was assumed for the years 2000–2099 A.D. (21^st^ century), which corresponds to a future with very rapid economic growth, global population peaks in mid-century and declines thereafter, a rapid introduction of new and more efficient technologies, and an energy system with no heavy dependence on one particular source^74^. Based on SST and atmospheric carbon dioxide (CO_2_) projections, shallow-water scleractinian coral cover loss due to bleaching was estimated from a combination of growth and mortality models^72^. Based on a projected increase in global temperatures of 2–4 °C over the coming century with a threshold for heat stress increasing by 0.1 °C every decade, the model suggested a coral cover decline of 25% to 75% for the main Hawaiian Islands by the end of the century^72,74^. We compared those modeled bleaching projections to existing coral reef data collected in 2000, which monitored bleaching events impacts in Fiji^73,75^. The modeled predictions of coral bleaching impacts were within the same range as recorded coral bleaching impacts in Fiji^73,75^. Given the similar levels of impact between Hawaiʻi and Fiji, it was deemed acceptable to transfer the projected bleaching impacts developed for Hawaiʻi to Kubulau. In our low and moderate bleaching scenarios, the current coral cover for reef areas shallower than 5 m was reduced by 10% and 30%, respectively. In our high bleaching scenario, to the current coral cover between 0–5 m and 5–10 m was reduced by 30% and 10%, respectively, because deeper waters are cooler and can reduce the impact of increased SST^76^. Given the large uncertainties and debate surrounding coral adaptation to heat stress^77^, these scenarios are not to be considered quantitative forecasts of percent coral cover change for this location, but instead conservative and large-scale probability-based estimates of the relative impact of predicted increases in SST and CO_2_ on coral reefs in Fiji over the next 100 years.

### Linked land-sea modeling framework

The land-sea framework is made of four key components: (1) InVEST SDR, (2) coastal sediment plume models, (3) marine drivers, and (4) coral reef predictive models. Sediment export (t.yr^−1^) was modeled at 30 m × 30 m resolution for each watershed using the InVEST SDR, based on the land-use from each scenario, topography, soil types, and rainfall data (Fig. 2c,d). The modeled sediment export was diffused from pour points representing stream mouths (Fig. 1) into the coastal zone using GIS distance-based models to generate the terrestrial driver grid data (60 m × 60 m) (t.yr^−1^) (Fig. 2e). Bathymetry and habitat maps, derived from remote sensing imagery, were coupled with GIS-based models, to generate marine driver grid data at 60 m × 60 m (geography and habitat) (Fig. 2f,g). The coral reef predictive models used Boosted Regression Trees (BRT) calibrated on local coral reef survey data and generated response curves representing the relationships of each individual environmental driver to each coral reef indicator, resulting in predictive maps of the benthic (% cover) and fish (kg.ha^−1^) indicators (60 × 60 m) (Fig. 2h,i). Once calibrated on local data, we applied this linked land-sea framework as a decision-support tool using scenario planning to identify coral reef areas vulnerable to sediment and climate change impacts, which we traced back to identify priority watersheds for forest conservation or restoration to promote coral reef resilience (Fig. 2i).

#### InVEST Sediment Delivery Ratio export models

For each scenario, we applied the InVEST SDR model^59^ to quantify the sediment export (t.yr^−1^) to the coast by watershed due to soil loss on hillslopes from overland erosion^59,60^ (Fig. 1c). SDR is spatially-explicit and operates at the resolution of the digital elevation model (DEM) input (30 m). For each cell, the model computes the amount of eroded sediment using the revised universal soil loss equation (RUSLE), and a sediment delivery ratio (SDR) to estimate the proportion of soil eroded on a given area that will travel to the stream mouth at the shoreline (see Hamel *et al*.^59^ for full details on the model). This approach was initially proposed by Borselli *et al*.^78^, which relies on modeling sediment transport throughout the landscape based on local topography, and therefore does not require hydrological modeling to determine the sediment ratio exported to the shoreline.

First, we estimated the overland gross erosion per cell using the empirical Revised Universal Soil Loss Equation (RUSLE) (*see* Equation 1)^79^:

1 $${Soil}\,{loss}={R}\times {K}\times {LS}\times {C}\times P\,$$

where R = rainfall erosivity (MJ.mm.ha^−1^.hr ^−1^), K = the rate of soil loss per rainfall erosion index unit, known as soil erodibility (ton-ha-hrs.MJ^−1^.ha^−1^.mm^−1^), LS = slope-length and gradient factor (derived from the DEM [30 × 30 m]^59^), C = a vegetation cover (C-factor) and P = management practice effectiveness (P-factor) (*see* Table S3 for more details on parameters). The map of rainfall erosivity was derived from monthly rainfall averages^80^ at a 100 × 100 m (P) and converted to erosivity using the Bols method applied in Indonesia (*see* Equation 2)^81^:

2 $${R}=\frac{2.5\times {{P}}^{2}}{100(0.073{P}+0.73)}$$

Soil erodibility (K), the rate of soil loss per rainfall erosion index unit^82,83^, was derived from the New Zealand Soil Survey dataset^84^, and used a value of K of 0.002 ton.ha.hr.MJ^−1^.ha^−1^.mm^−1^ to fill in missing values that were not available in the tables. C-factors derived from existing literature and studies conducted in similar regions were assigned to each land cover/use type (see Table S3 for more details on parameters)^82,85–88^. Management practice effectiveness (P factor) was not considered in this model due to lack of data^59^.

Then, the SDR model estimated the proportion of soil eroded on a given area that travelled to the stream mouth at the shoreline. The SDR model is based on the concept of hydrological connectivity to estimate sediment retention and export to the shoreline (see Borselli *et al*.^78^ for more details). First, the SDR computes a connectivity index (IC_i_) for each pixel *i* based on the upslope area and downslope flow path^78^. A streamflow accumulation threshold was set to define streams based on the DEM^59^. Given the lack of empirical data for the region, the connectivity of the model was verified by comparing predicted stream outputs to available stream maps. Sub-watersheds were created using the Basins function in ArcGIS and pour points at the shoreline were edited for accuracy in comparison to satellite imagery and bathymetry data^89,90^. Then, a sediment delivery ratio was derived for each pixel *i* based on the connectivity index^59^. The SDR model parameters include an IC_0_, a Borselli k-factor, and a maximum allowable SDR that define the shape of the relationship between the SDR and the connectivity index (IC_i_)^59^. The calibration parameters IC_0_ and the k-factor were set to 0.5 and 2.0, respectively, and the maximum allowable SDR was set to 0.8^59^ (see Sharp *et al*.^58^ for more details on effects of parameterization). This approach was selected since it requires a minimal number of parameters and is spatially-explicit. We note that the models have yet to be quantitatively validated against local datasets and were parameterized with values from other regions, which can differ in terms of climate and soil conditions^91^.

#### Coastal sediment plume models

To link the sediment and coral reef models, we modeled the impact of sediment runoff on coastal water quality. In order to do so, we generated a water quality map (60 × 60 m) representing the total sediment load (represented by TSS) from all the watersheds discharging into Kubulau coastal waters, for each land-use scenario. First, the modeled sediment export from each watershed was diffused into coastal waters by adapting a previously developed dispersal plume model in ArcGIS to represent the point source nature of stream discharge in local coastal water conditions^53,57,92^ (Fig. 2d). To do so, we created a cost-path surface (*c*) that quantifies the least accumulative cost-distance (impedance) of moving planimetrically through each cell from each pour point using a composite of three marine drivers known to affect diffusion: depth (m), distance to stream mouth (m), and wind exposure (degree) (*see* ‘Marine drivers models’ section below for more details)^11,57,93^. Then, the spread of sediment into coastal waters from each pour point was modeled using a decay function, which assigned a portion of the remaining quantity from the previous cell in all adjacent cells, based on the cost-path surface until a maximum distance of 3 km from the shoreline was reached^53,57^ (*see* Equation 3). This threshold was based on the distance between river mouths measured in ArcGIS and locations where sediment impacts on coral reefs have been recorded in the past^94^.

3 $${{S}}_{{i}}={{s}}_{{p}}\times {{e}}^{-{{c}}^{2}/{{D}}_{{c}}}$$

where *S*_*i*_ = cell value for dispersed sediment (t.yr^−1^) for watershed *i*, *S*_*p*_ = Sediment load (t.yr^−1^) at each watershed pour point (obtained from summarizing the total sediment export per watershed), *c* = cost-path surface (unitless), *D*_*c*_ = cost-path surface threshold distance from the shore for each decayed sediment plume per watershed (equivalent to 3 km from the shoreline). Last, we summed all the individual watershed sediment plume gridded maps in ArcGIS to obtain the total sediment load (represented by TSS) per land-use scenario for each pixel of coral reef area. This approach to modeling coastal sediment discharge is diffusive, and thus allows for wrap around coastal features, but does not account for nearshore advection that acts to push suspended sediment in specific directions^53^. We used these diffusive models to derive conservative estimates of sediment plumes, since the nearshore circulation patterns were unknown for our study site.

#### Coral reef indicators

To measure coral reef resilience, we considered the percent cover of four benthic groups (crustose coralline algae [CCA], scleractinian corals, turf algae and macroalgae) and the biomass of four fish groups (kg.ha^−1^) based on their ecological roles and cultural importance to local communities^62^ (*see* Table S1 for more details). The benthic indicators are known to respond to changes in land-based runoff, which influence the distribution of fish taxa^11,40^, and therefore support aspects of coral reef ecological resilience^13,65^. Fish taxa identified as important for subsistence and cultural practices by the local communities were modeled according to their ecological role: (1) browsers, (2) grazers, (3) scrapers, and (4) predators (*see* Tables S1 and S2 for more details)^62,65^. We derived percent cover of the benthic indicators (%) and biomass of the fish indicators (kg.ha^−1^) from reef survey data collected by the Wildlife Conservation Society (WCS). The field dataset comprised 163 survey locations randomly stratified by depth (deep [12–15 m], shallow [5–8 m], top [0.5–2 m]), habitat (forereef and backreef areas), and management (open or closed to fishing) (Fig. 1), collected over three sampling periods (2009–2010) (*see*^64,95^ for more details).

#### Marine driver models

The marine driver grid maps (60 × 60 m) were derived from bathymetry^96^ and benthic habitat^95^ maps for the site using GIS-based tools (Fig. 2f,g)^97,98^. Based on existing literature and local community input, local geography (depth and distance to shore) and habitat characteristics (topography, complexity, and exposure) were identified as important drivers of the modeled coral reef benthic and fish indicators (*see* Table S4 or *see* Stamoulis & Delevaux *et al*.^97^ for more details on processing methods). Depth and distance from shore were used to account for variation arising from spatial location. Depth was derived from bathymetry at 4 m resolution^96^ using passive remote sensing techniques, and distance from shore was calculated as the Euclidean distance (m) from the coastline, using the national coastline map in ArcGIS^99^. Three types of habitat drivers, representing direct and indirect effects of seafloor topography on benthic and fish communities were also derived from this bathymetry data^96^: (1) habitat topography, (2) habitat complexity, and (3) habitat exposure. Habitat topography metrics, represented by Bathymetric Position Index (BPI) and slope, described the position of the reef relative to the surrounding area. These metrics were computed using the Benthic Terrain Modeler tool for two neighborhood sizes (60 and 240 m radii) to capture habitat topography at different spatial scales^100–102^. Habitat complexity metrics that describe fine-scale topographic structure, represented by slope of slope, planar curvature, and profile curvature were computed using the DEM Surface and Curvature Tools in ArcGIS^96,98^. Habitat exposure metrics were used to characterize the direct and indirect effects of water flow due to fine-scale seafloor topography and directionality. These metrics were derived by computing seafloor aspect, the steepest downslope direction of the seafloor measured in degrees, using the Aspect tool in ArcGIS^96,98^. We estimated exposure to winds by calculating the circular mean and standard deviation of aspect and converted the circular mean into measures of northness and eastness using the sine and cosine functions, respectively, from the Spatial Analyst toolbox in ArcGIS^96,98^. Four types of habitat connectivity metrics, representing direct and indirect effects of habitat composition and fragmentation on benthic and fish communities, were derived from the benthic habitat map, with a 10 m minimum mapping unit in FRAGSTATS software: (1) contiguity, (2) fractal dimension, (3) proximity, and (4) Shannon diversity index^95,103^. We note that it is not necessary to generate all these drivers in order to apply this method elsewhere, it depends on whether a bathymetry and/or habitat map is available.

#### Spatial predictive coral reef models

We used BRT to build the coral reef models (Fig. 2h)^104^. Tree-based models are effective at modeling nonlinearities, discontinuities (threshold effects), and interactions between variables, which is well suited for the analysis of complex ecological data^105–107^. BRT models can accommodate many types of response variables, including presence/absence, count, diversity, and abundance data^108^. Since the coral reef indicators were all continuous variables, the response variables were modeled using a Gaussian (normal) distribution, and appropriate data transformations (square root for benthic indicators and fourth root for fish biomass) were applied to improve the normality of the response variable distributions. The highly correlated (r > 0.7) environmental drivers were removed from the coral reef models^97^. We calibrated the BRT models on coral reef data to determine the most influential drivers (among the simultaneously tested predictors) and estimate the underlying relationships between the modeled indicators and the key drivers using response curves^108,109^. The values of the terrestrial and marine drivers’ grid maps were sampled using bilinear interpolation at the location of each reef survey (start of the transect) in ArcGIS. This approach takes a weighted average of the 4 nearest cell values, thereby accounting for the relative position of the reef surveys on the predictor grids and their different native spatial scales.

A BRT model was independently developed for each coral reef indicator to determine the relative influence of terrestrial and marine drivers using the values of the coral reef indicators and interpolated terrestrial and marine driver values at the reef survey locations. First, we calibrated the benthic indicators’ models as a function of the terrestrial and marine drivers. Then, we calibrated the fish indicators’ models as a function of the terrestrial and marine drivers, as well as the empirical abundance of benthic groups as additional predictors in the models for the fish groups. The calibration process used an internal ten-fold cross-validation to maximize the model fit and determine the optimal combinations of four parameters: (1) learning rate (lr), (2) tree complexity (tc), (3) bag fraction (bag), and (4) the maximum number of trees (*see* Elith *et al*.^108^ for more details). We used the percent deviance explained (PDE) and internal ten-fold cross validation PDE (CV PDE) as performance measures of the model optimum. The optimal models explained the most variation in the response variables (i.e., greatest CV PDE) and were selected as the best and final models. The model calibration was conducted in R software using the gbm package^108,110,111^. Spatial autocorrelation of the response variables was tested using Moran’s I Index for both the raw values and the ecological model residuals^112^.

Using the calibrated coral reef models, we predicted and mapped the distribution of each coral reef indicator on a cell-by-cell basis using the values of the terrestrial and marine drivers at each grid cell across the coral reef model domain. Predictive maps were generated for each indicator under present conditions and future scenarios (land-use change, climate change, and combined scenarios), described in more detail below (Fig. 2a,b)^72,73^. The coral reef predictive maps were generated at 60 × 60 m to account for the dimensions of the reef survey methods (i.e., 25 m transects), the transect bearings, and the positional accuracy of global positioning system used to navigate to them in the field^113,114^. The boundaries of the coral reef model domains comprised the *qoliqoli* boundaries to capture the spatial extent of this management unit and the offshore boundary corresponded to the maximum surveyed depth (i.e., 22 m) (Fig. 1b). First, we spatially predicted each benthic indicator as a function of its key drivers. Then, we spatially predicted the fish indicators as a function of their key drivers, including the predicted distribution of the benthic indicators.

In order to evaluate the quality of the coral reef model predictions, we compared the measured and predicted values of the coral reef indicators under present conditions. The values of the interpolated predictions and surveyed coral reef indicators at these locations were compared with a linear regression (R^2^ and p-value). The predicted values of the benthic and fish grid maps were sampled using bilinear interpolation at the location of each reef survey (start of the transect) in ArcGIS, thereby accounting for the relative position of the reef surveys on the predicted grids. Then, we overlaid the predicted maps with the survey point values for each indicator using the same color ramp scale for the legend to enable visual comparison. The spatial predictions were performed in the R software using the dismo and raster packages^111,115,116^.

### Coral reef scenario impact assessment

We identified coral reef areas that could be impacted by land-use and/or climate change scenarios. To do so, we calculated differences between predictions of coral reef indicators under the land-use change, climate change, and combined scenarios, compared to present conditions. We computed the significance of the pairwise differences per grid cell for each coral reef indicator relative to the mean and variance of all differences across the coral reef model domain using the SigDiff function from the R package SDMTools^117^. The grid cells representing significant differences (α = 0.10) were reclassified to indicate where predictions were significantly different than present conditions under each scenario^34^. For the combined scenarios, we overlaid the coral reef areas of significant differences under land-use and climate change scenarios and delineated areas of overlap, where both drivers could potentially interact. These areas were combined into a single map to display the spatial pattern of potential impact per scenario. Finally, the areas of significant differences for each coral reef indicator were used to quantify the relative changes in benthic habitat and fish biomass within those areas. For areas where coral bleaching and sediment runoff overlapped, we summed the changes in abundance of each coral reef indicator. Although interactions between sediment runoff and bleaching were not explicitly modeled, this approach allowed us to delineate coral reef areas where cumulative impacts and interactions could occur. High turbidity and suspended sediment can mitigate elevated SST and associated bleaching impacts (i.e., antagonistic effect) but these interactions remain poorly understood^16,17^. Due to lack of data, nutrient runoff was not explicitly modeled in this study, but nutrients are known to bind and travel with sediment, thereby potentially contributing to lack of recovery from bleaching through promoting algae growth (i.e., synergistic effect)^14,15^. However, given the limited understanding of these complex processes and the lack of data to represent nutrient runoff, we assumed that these effects were additive. Therefore, we applied the precautionary principle and considered these coral reef areas as vulnerable to cumulative impacts^118^.

### Spatial prioritization of conservation

In order to locate the most effective areas to prioritize forest conservation (i.e., prevent deforestation) and restoration to foster coral reef resilience, we linked the coral reef areas vulnerable to sediment runoff from land areas within upstream watersheds that contributed the major portion of the sediment load to those areas. Using the individual watershed sediment plume maps (60 m × 60 m) from the coastal plume models, we identified the watersheds contributing the majority of sediment runoff to coral reef areas likely to change under land-use and/or climate change scenarios, compared to present conditions. To do so, we linked the coral reef areas showing significant difference to the watersheds upstream, which contributed the majority (>90%) of the total sediment load (represented by TSS) at those areas in R and ArcGIS. The linked coral reef areas and watersheds were combined into a single map to display the land-sea linkages for the land-use and climate change scenarios. Then, using the sediment export maps (30 m x 30 m) from the SDR models, we identified land areas contributing the most sediment runoff to coral reef areas likely to change under land-use and/or climate change scenarios, compared to present conditions. For climate change scenarios, we identified priority land areas in the linked watersheds contributing a large portion (>66%) of sediment export to downstream coral reefs under current land-use. For the land-use change scenarios, we calculated the significant differences in sediment export in the linked watersheds per grid cell, compared to present conditions using the SigDiff function^117^. For each scenario, the grid cells representing significant differences were reclassified to indicate where sediment export was significantly different from present conditions (α = 0.10). In ArcGIS, we created a 100 m buffer around those priority land areas based on conservation and logging best management practices^60,119^. To visually represent those results, the linked watersheds were combined into a single map to display where conservation and/or restoration actions could foster coral reef resilience based on the land-use and climate change scenarios and overlaid the priority land areas for each scenario.

### Data availability statement

All the data produced in this study is available from the data repository fisgshare: 10.6084/m9.figshare.6823508.

## Results

### Spatial variation of terrestrial and marine drivers under present conditions

**Figure 3:**
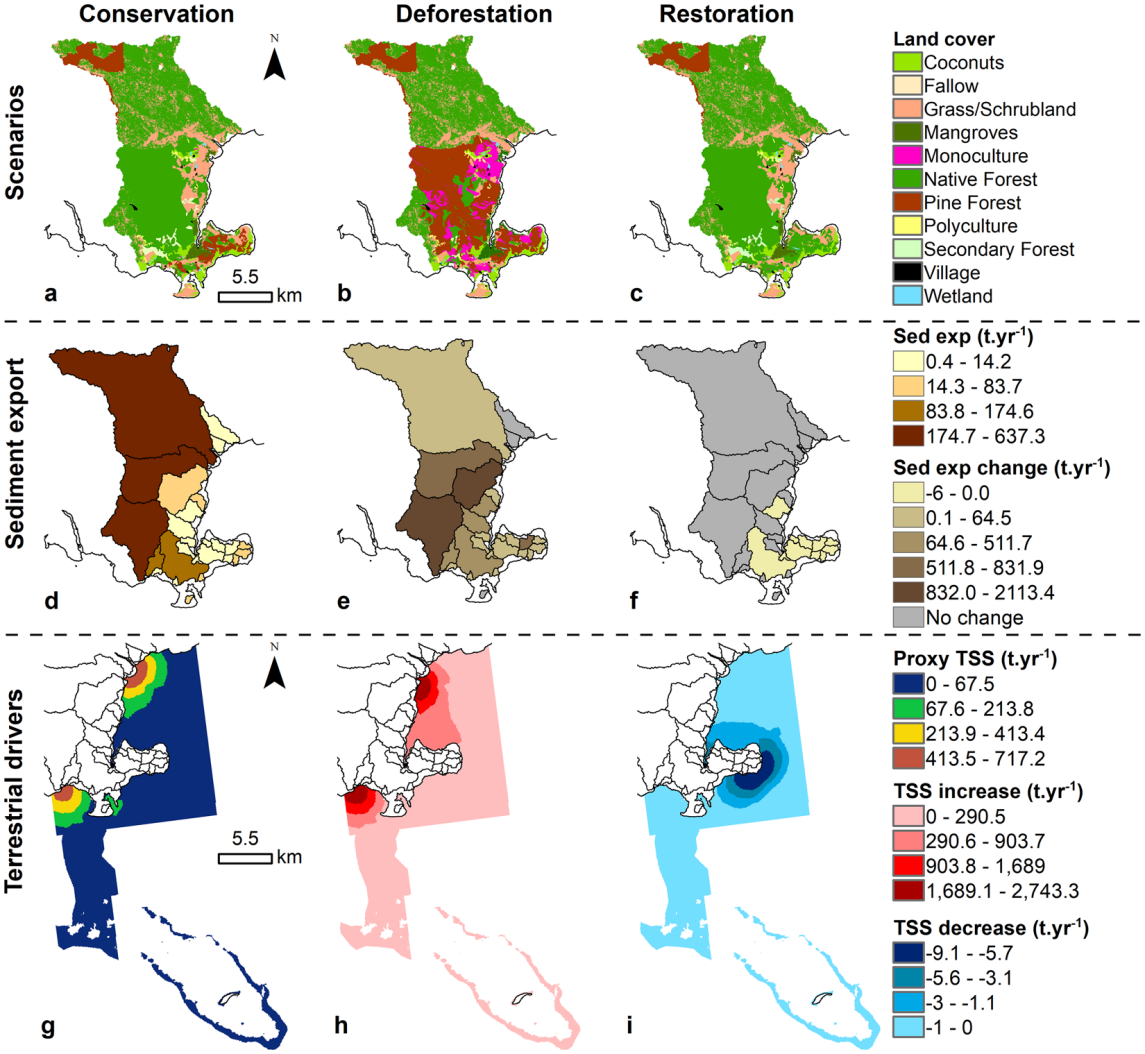
Land-use with associated sediment export and coastal plumes under different forest management scenarios. (**a**) Conservation/present; (**b**) Deforestation; (**c**) Restoration scenarios with their corresponding **(d**) sediment load or (**e**,**f**) change in sediment load compared to present, and (**g**) coastal sediment discharge or (**h**,**i**) change in sediment discharge compared to present.

**Figure 4:**
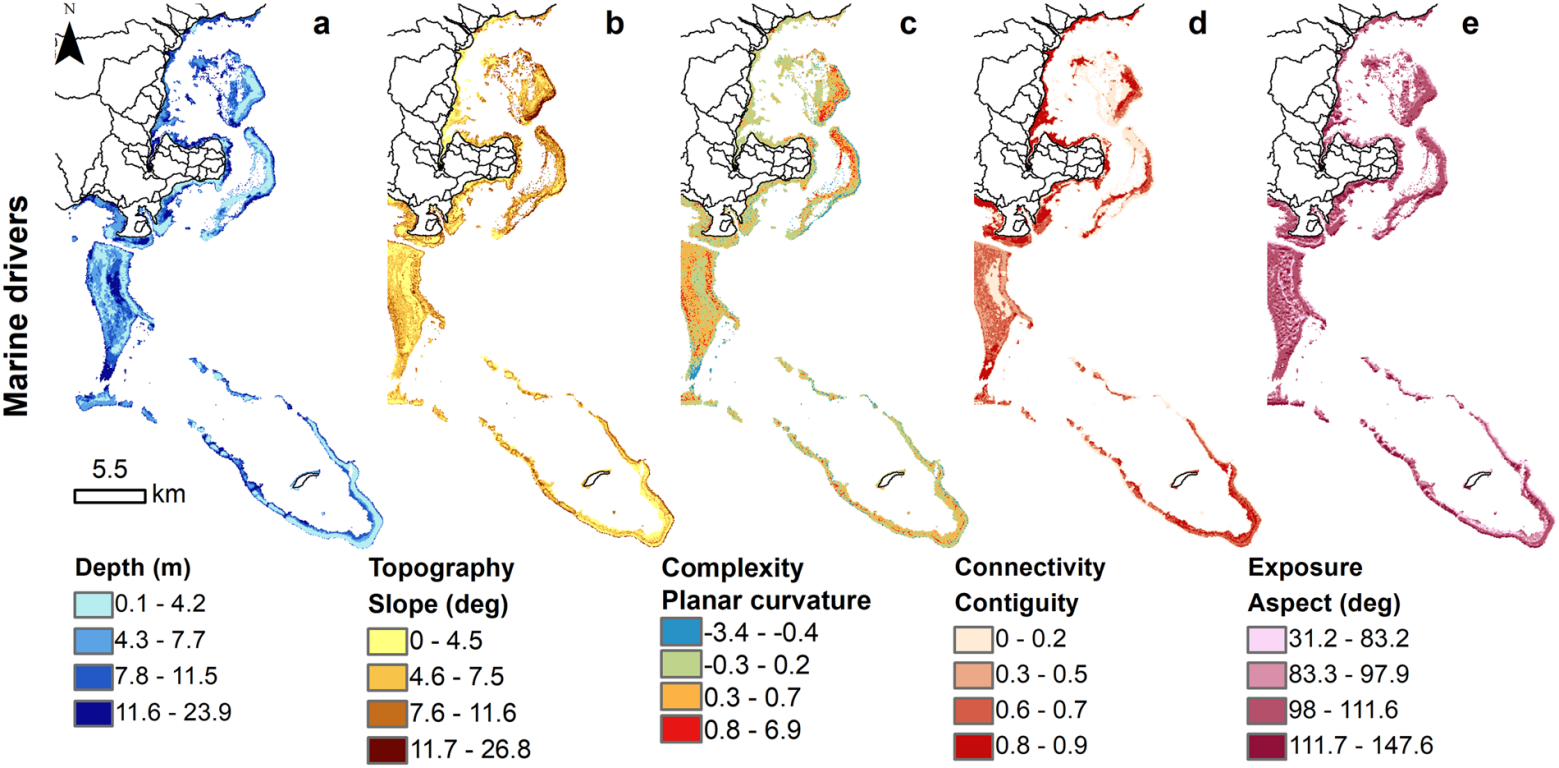
Marine drivers of coral reefs. The marine drivers are represented by (**a**) depth (m), (**b**) habitat topography (slope [degree]), (**c**) habitat complexity (planar curvature [standard deviation]), (**d**) Habitat connectivity (contiguity), and (**e**) habitat exposure (surface aspect [degree]).

Currently, the majority of Kubulau’s land-use is made up of native forest (13,958.9 ha), shrubland (3,277.4 ha), and secondary forest (306.2 ha), with some pine plantations for logging (573.8 ha), and small amount of agricultural land devoted to monoculture of agricultural crops (e.g., taro [1.2 ha] and kava [2.3 ha]) (Fig. 3a). Correspondingly, the sediment export model under the conservation scenario with existing land-use resulted in a total sediment export of 2,142.7 t.yr^−1^ (or 10.3 t.km^-2^), with three large watersheds discharging approximate a total of 1,501.8 t.yr^−1^ and contributing over 70% of the sediment load (Fig. 3d). Consequently, the terrestrial driver (represented by TSS) showed higher values of suspended sediment in the southern and eastern bays of Kubulau District (Fig. 3g). In terms of the marine drivers, the depth of the marine habitats ranged from 0.1 to 23.9 m, with habitats along the reef slopes of the barrier reefs being topographically more complex, while habitats nearshore and in the lagoon were more contiguous and less exposed (Fig. 4a–e).

### Coral reef predictive models

**Figure 5:**
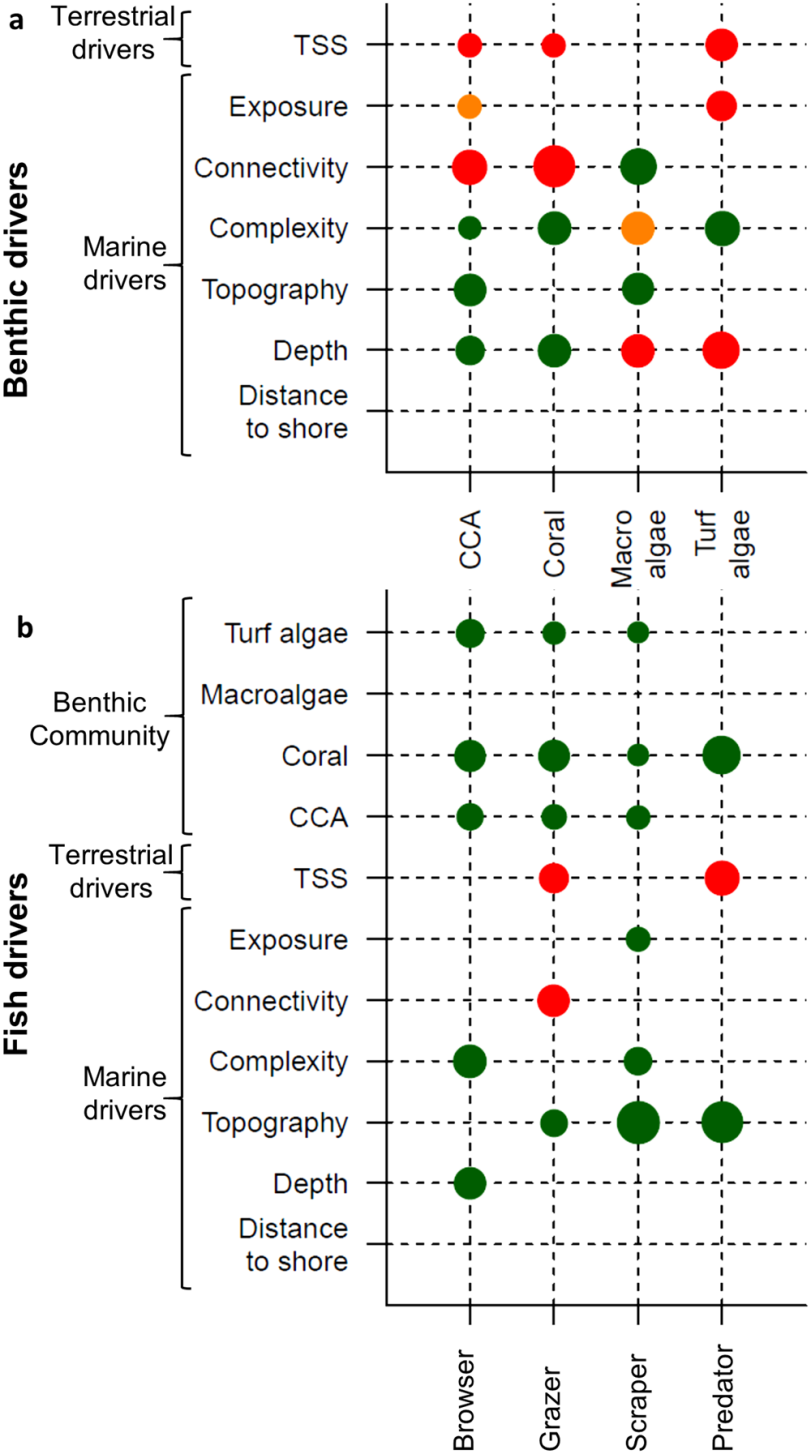
Coral reef models. Benthic (top) and fish (bottom) indicators are represented along the X axes and the terrestrial and marine drivers on the Y axes. The bubble size represents the relative percent contribution of each driver and the color indicates whether the relationship between the indicator and driver is positive (green), convex/concave (orange), or negative (red).

The PDE of the final coral reef models accounted for 29.7–81.5% and the internal CV PDE accounted for 12–50.5% (*see* Table S5 for more details). Analysis of the residuals from the final coral reef models showed no spatial autocorrelation (Moran’s I Index p > 0.1). In terms of the terrestrial drivers, the coral reef models identified that sediment runoff (represented by TSS) was a key driver of coral reef state (Fig. 5). TSS had a negative effect on CCA, coral, and turf algae cover, and grazer and predator biomass. In terms of the marine drivers, the coral reef models identified depth, habitat topography and complexity as key drivers of coral reef state (Fig. 5). The coral reef models also showed that the benthic groups (CCA, coral, and turf algae) were key drivers of the fish community (Fig. 5). All the fish indicators were strongly associated with corals, and herbivores were also positively associated with CCA and turf algae. CCA and corals were positively associated with depth, while macroalgae and turf were negatively associated with depth. Most benthic and fish indicators were positively associated with steeper, deeper reef slopes and more complex habitat, except for macroalgae, which was negatively associated with habitat complexity.

### Spatial predictive maps of coral reef indicators

**Figure 6:**
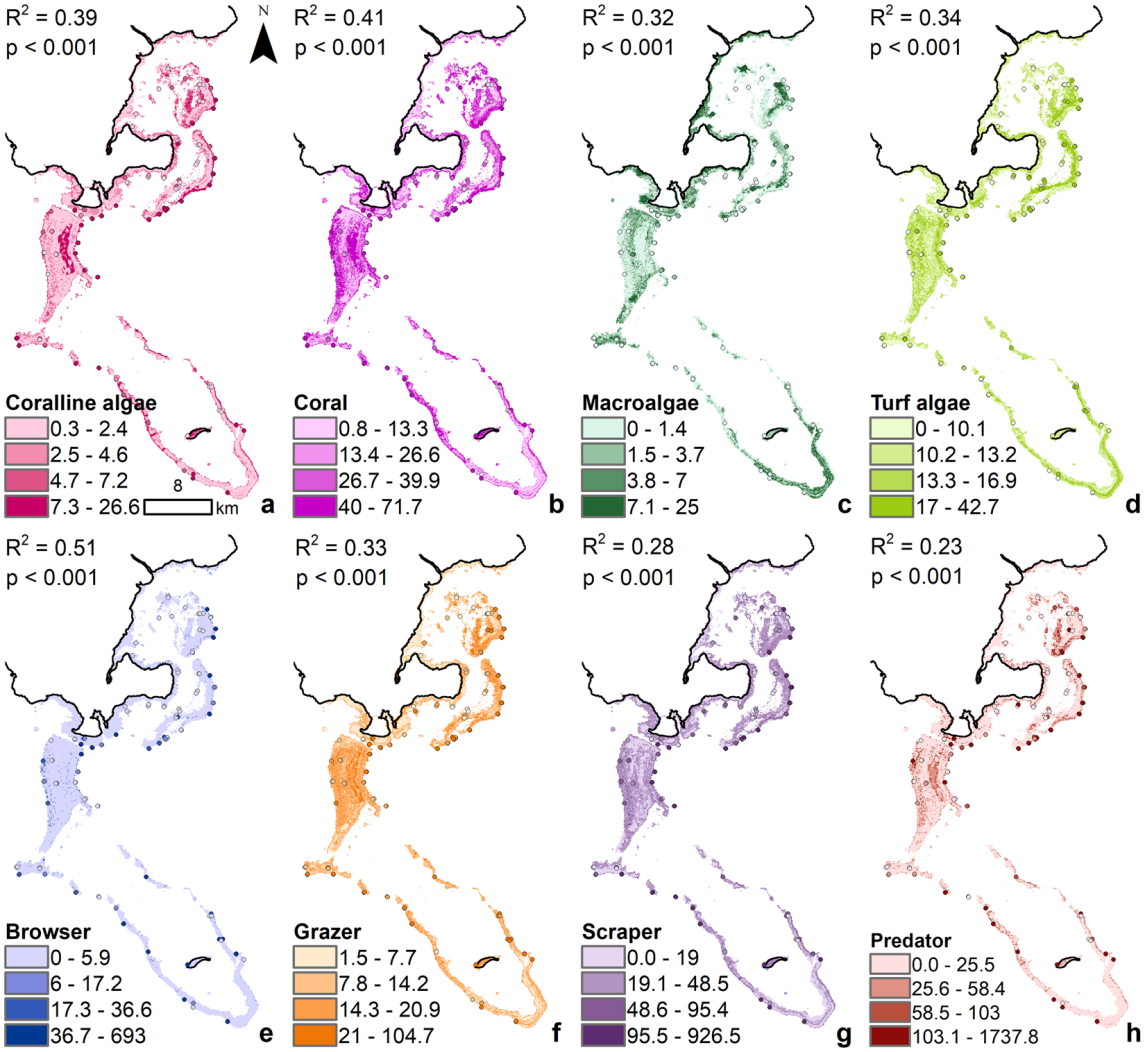
Present coral reef indicators. (**a**–**d**) Benthic indicators are measured in % cover and (**e**–**h**) fish indicators are measured in kg.ha^−1^. The predicted maps are overlaid with the survey points using the same color ramp for visual comparison, combined with the R^2^ and p-values.

The comparison of the predicted coral reef indicator values and empirical survey values showed low R^2^ (<0.4), but statistically significant relationships (*see* Fig. S9 for more details). The R^2^ was higher for CCA and coral predictions compared to turf and macroalgae predictions, while the browsers and grazers predictions performed better than the scrapers and predators. Under present conditions, the coral reef models predicted a benthic community with low CCA cover ($\bar{X}$ = 3.0%; range = 0.3–26.6%) and high coral cover ($\bar{X}$ = 26.7%; range = 0.8–71.7%), particularly along the reef slopes, with macroalgae cover low ($\bar{X}$ = 2.6%; range = 0–25%) and more concentrated inshore and turf algae cover ($\bar{X}$ = 12.8%; range = 0–42.7%) higher on the reef flats (Fig. 6). The coral reef models predicted a fish community with many scrapers ($\bar{X}$ = 26.9 kg.ha^−1^) and predators ($\bar{X}$ = 33.2 kg.ha^−1^) and few browsers ($\bar{X}$ =0.96 kg.ha^−1^) and grazers ($\bar{X}$ = 12.2 kg.ha^−1^), with higher fish biomass for all indicators in more complex habitat with high CCA, coral, and turf algae cover. When predictions for benthic cover and fish biomass under present conditions were compared to benthic and fish survey data at the site level, BRT predictions consistently underestimated means of empirically measured values. However, the BRT predictions well represented the relative differences in mean abundance between indicators for the entire study site (CCA: 6.8%, coral: 38.6%, macroalgae: 2.5%, turf algae: 13.4%, browser: 33.6 kg.ha^−1^, grazer: 21.9 kg.ha^−1^, scraper: 104.7 kg.ha^−1^, predator: 124.1 kg.ha^−1^).

### Coral reef impact assessment

**Figure 7:**
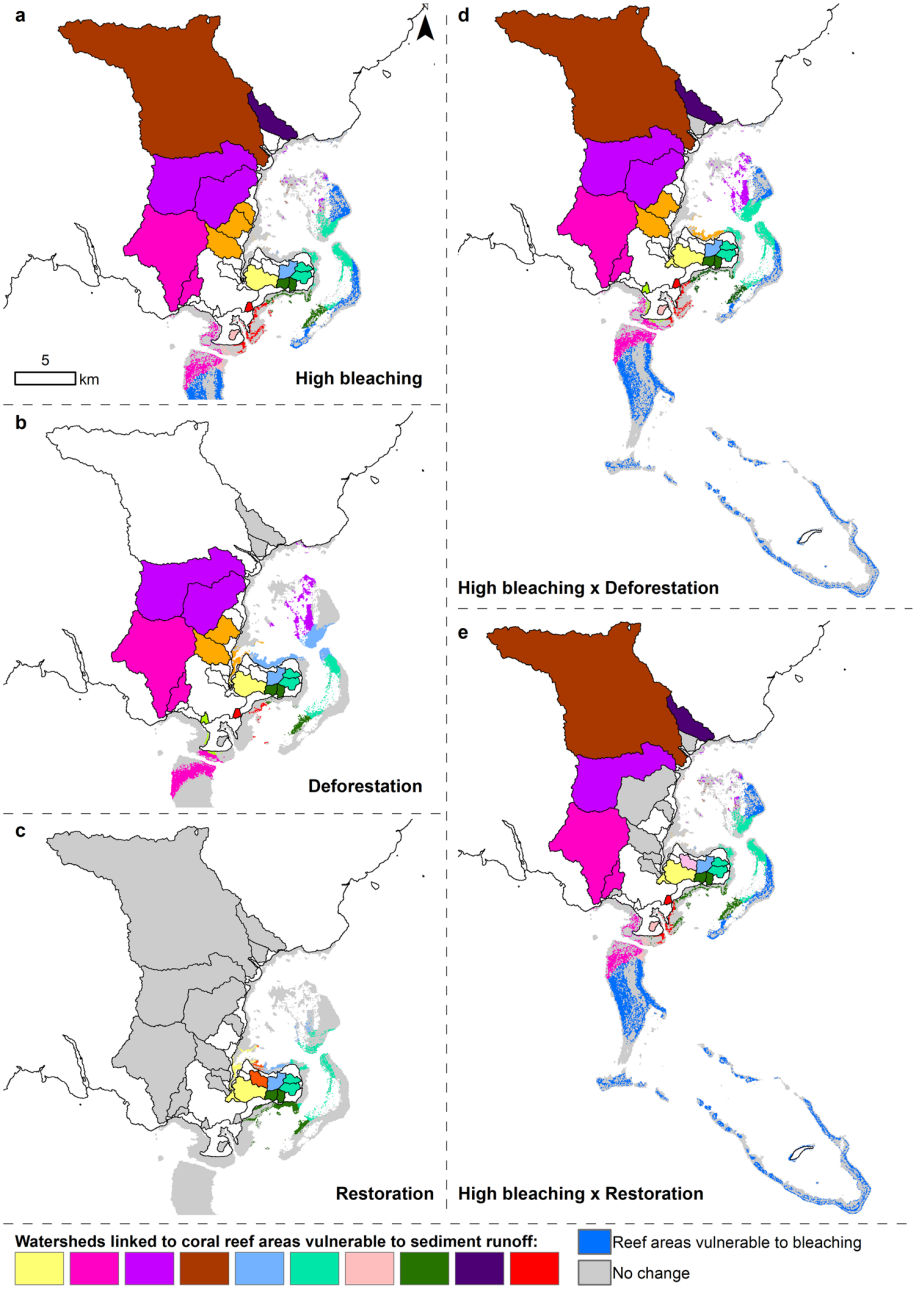
Key land-sea linkages. Watersheds linked to coral reef areas are indicated by matching colors under each scenario. (**a**) Under the current land-use and high climate change scenario, watersheds are linked to coral reef areas vulnerable to sediment runoff and bleaching impacts. (**b**) Under the deforestation scenario, watersheds are linked to coral reef areas vulnerable to their sediment runoff. (**c**) Under the restoration scenario, watersheds are linked to coral reef areas with high recovery potential due to reduced sediment runoff. (**d**) Under the deforestation and high climate change scenario, watersheds are linked to coral reef areas vulnerable to their sediment runoff and bleaching impacts. (**e**) Under the high bleaching and restoration scenario, watersheds are linked to coral reef areas with high recovery potential from reduced sediment runoff under restoration and coral reef areas vulnerable to sediment runoff under impact from high bleaching.

Under the climate change scenarios (low, moderate, and high) and present land-use, the coral reef models predicted a decrease of 1,389–1,897 ha in coral cover and 13.4–69.5 t of fish biomass, with an average loss ranging from 3–9.4% coral cover and 17.7–40.1 kg.ha^−1^ fish biomass (Table 1). The potential bleaching impacts are higher on the offshore coral reef areas, while the back reef areas located further away from the land are susceptible to both bleaching and sediment runoff (Fig. 7a). Under the deforestation scenario, monoculture of agricultural crops (taro and kava) and pine plantations were expanded by 1,565.7 ha and 4,724.7 ha, respectively, resulting in a loss of 5,192.3 ha of native forest, 268.4 ha of secondary forest, and 825.8 ha of shrubland (Fig. 3c). Under deforestation, the sediment models indicated an additional 7,441 t.yr^−1^ of sediment discharging at the shoreline (i.e., total sediment load = 9,583.8 t.yr^−1^), resulting in an increase of TSS in the southern and eastern bays of Kubulau District (Fig. 3e). Consequently, the coral reef models predicted a coral cover decrease of over 1,940 ha and 60.6 t of fish biomass, with an average loss of 3.3% coral cover and 43.9 kg.ha^−1^ fish biomass (Table 1). The potential impact to coral reefs is larger in the south and south east region of the site (Fig. 7b). Under the restoration scenario, 573.8 ha of pine plantations were restored to native forest (Fig. 3e), the sediment models indicated a decrease of 24.2 t.yr^−1^ of sediment (i.e., total sediment load = 2,118.5 t.yr^−1^), compared to current sediment export, resulting in a decrease in TSS around the southeast coastal areas of Kubulau District (Fig. 3f). Consequently, the coral reef models predicted coral cover to increase in 577 ha of benthic habitat along with a 5.7 t increase of fish biomass, with an average increase of 1.1% coral cover and 13.9 kg.ha^−1^ fish biomass (Table 1). The potential recovery of coral reefs is larger in the south and south east region of the site (Fig. 7c).

**Table 1:** Coral reef impact assessment.

Scenarios	CCA	COR	MAC	TUR	BROW	GRAZ	SCRP	PISC	Total		
	ha (%)	ha (%)	ha (%)	ha (%)	tonnes (kg/ha)	tonnes (kg/ha)	tonnes (kg/ha)	tonnes (kg/ha)	area (ha)	tonnes (kg/ha)	ws (#)
Deforestation	184 (−0.3)	708 (−3.3)	0.0 (0.0)	1048 (−1.7)	−2.2 (−8.3)	−9.3 (−4.0)	−6.1 (−4.1)	−43.0 (−27.5)	1940	−60.6 (−43.9)	14
Restoration	129.6 (0.2)	282 (1.1)	0.0 (0.0)	165 (1.2)	0.2 (3.8)	0.9 (0.9)	0.5 (1.5)	4.0 (7.7)	577	5.7 (13.9)	8
Bleaching Low	0.0 (0.0)	1389 (−3.0)	0.0 (0.0)	0.0 (0.0)	−0.1 (−3.4)	−1.8 (−1.5)	−1.1 (−0.8)	−10.4 (−12.0)	1389	−13.4 (−17.7)	14
Bleaching Moderate	0.0 (0.0)	1389 (−9.1)	0.0 (0.0)	0.0 (0.0)	−0.1 (−3.6)	−7.3 (−3.1)	−2.5 (−1.6)	−16.7 (−12.6)	1389	−26.6 (−20.9)	14
Bleaching High	0.0 (0.0)	1897 (−9.4)	0.0 (0.0)	0.0 (0.0)	−4.9 (−10.4)	−11.6 (−3.1)	−10.1 (−5.2)	−42.9 (−21.3)	1897	−69.5 (−40.1)	17
Bleaching Low × Deforestation	184 (−0.3)	1339 (−3.7)	0.0 (0.0)	1048 (−1.7)	−2.2 (−8.3)	−9.8 (−4.1)	−6.4 (−4.2)	−45.6 (−27.5)	2570	−64.0 (−44.1)	18
Bleaching Mod × Deforestation	184 (−0.3)	1662 (−8.1)	0.0 (0.0)	1048 (−1.7)	−2.2 (−8.3)	−13.5 (−4.2)	−6.9 (−4.4)	−47.6 (−28.5)	2894	−70.3 (−45.4)	18
Bleaching High × Deforestation	184 (−0.3)	2145 (−8.8)	0.0 (0.0)	1048 (−1.7)	−6.4 (−11.6)	−14.6 (−4.4)	−14.4 (−6.1)	−59.5 (−32.7)	3377	−94.9 (−54.8)	18
Bleaching Low × Restoration	129.6 (0.2)	104 (1.7)	0.0 (0.0)	165 (1.2)	0.2 (4.0)	0.8 (1.1)	0.5 (1.7)	3.8 (9.3)	399	5.3 (16.1)	15
Bleaching Mod × Restoration	129.6 (0.2)	104 (1.7)	0.0 (0.0)	165 (1.2)	0.2 (4.0)	0.8 (1.1)	0.5 (1.7)	3.8 (9.3)	399	5.4 (16.1)	15
Bleaching High × Restoration	0.0 (0.0)	107 (1.7)	0.0 (0.0)	165 (1.2)	0.2 (4.0)	0.8 (1.1)	0.5 (1.7)	4.2 (13.0)	402	5.7 (19.7)	15
Bleaching Low × Restoration	0.0 (0.0)	1351 (−3.1)	0.0 (0.0)	0.0 (0.0)	−0.1 (−3.6)	−1.8 (−1.5)	−1.0 (−1.3)	−10.5 (−12.0)	1351	−13.5 (−18.4)	9
Bleaching Mod × Restoration	0.0 (0.0)	1400 (−9.1)	0.0 (0.0)	0.0 (0.0)	−0.1 (−3.6)	−7.2 (−3.1)	−2.5 (−1.7)	−16.7 (−12.7)	1400	−26.7 (−21.2)	9
Bleaching High × Restoration	0.0 (0.0)	1906 (−10.2)	0.0 (0.0)	0.0 (0.0)	−11.5 (−10.5)	−11.5 (−3.1)	−10.1 (−5.2)	−42.4 (−21.4)	1906	−75.5 (−40.2)	9

The total significantly different area (ha) and associated average % cover change are reported for the benthic indicators per scenario relative to present conditions. The total biomass change (tonnes or t) and associated average loss and/or gain (kg.ha^−1^) over significantly different areas are reported for the fish indicators per scenario relative to present conditions. Number of watersheds (ws) where priority areas for conservation or restoration have been identified.

**Figure 8:**
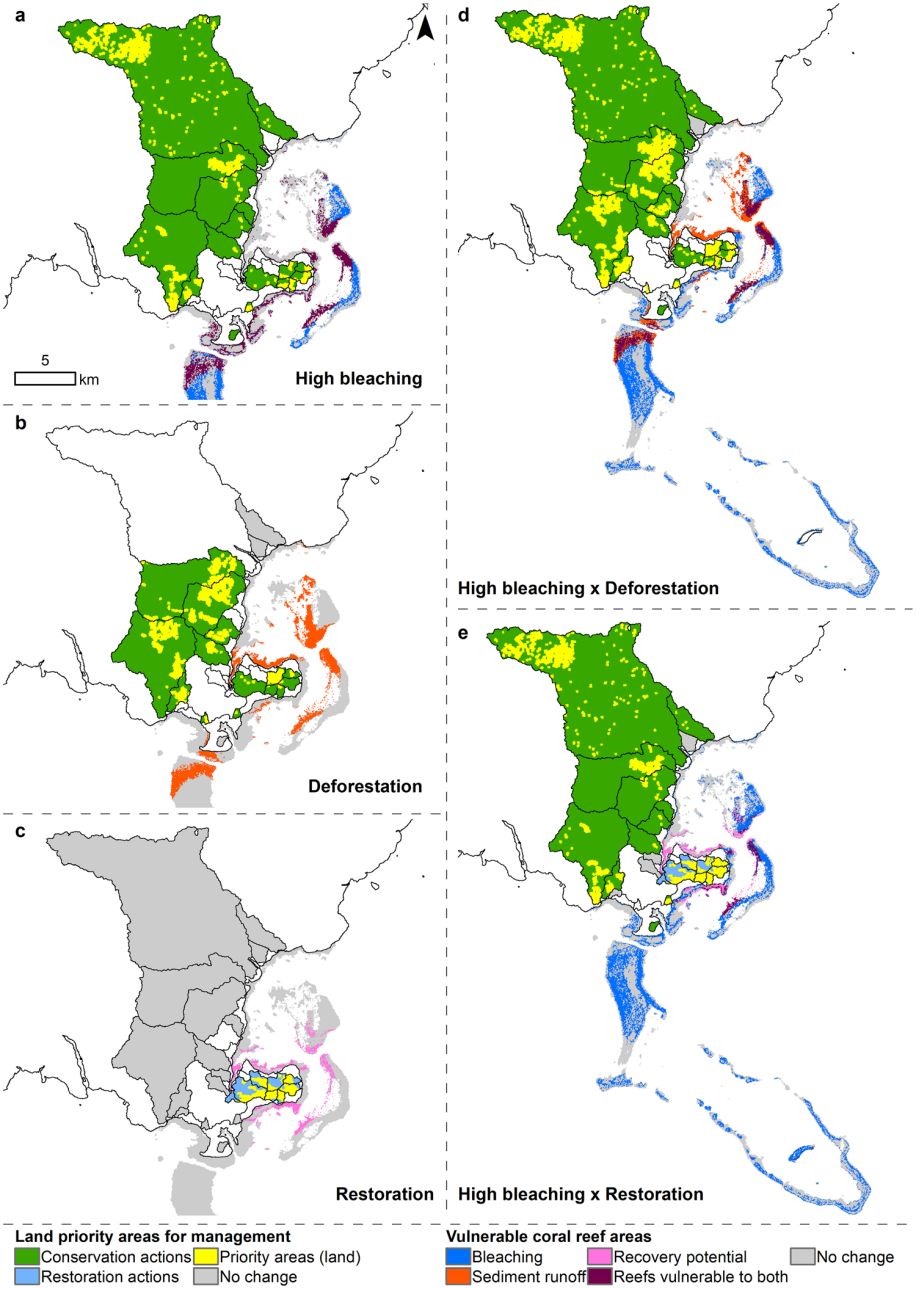
Land priority areas for management actions and coral reef areas vulnerable to human impacts. Priority watersheds where conservation (i.e., avoiding deforestation) or restoration can benefit coral reef areas are indicated in green or blue, respectively, and priority areas within watersheds are shown in yellow. (**a**) Under the present land-use and high climate change scenario, watersheds linked to coral reef areas vulnerable to bleaching and sediment runoff, contain priority land areas for conservation. (**b**) Under the deforestation scenario, watersheds linked to coral reef areas vulnerable to their sediment runoff, contain priority land areas for conservation. (**c**) Under the restoration scenario, watersheds linked to coral reef areas with high recovery potential, contain priority land areas for restoration. (**d**) Under the deforestation and high climate change scenario, watersheds linked to coral reef areas vulnerable to their sediment runoff and bleaching impacts, contain priority land areas for conservation. (**e**) Under the high bleaching and restoration scenario, watersheds linked to coral reef areas with high recovery potential under restoration and coral reef areas vulnerable to sediment runoff under impact from high bleaching, contain priority land areas for conservation and/or restoration.

When combining deforestation with climate change (low, moderate, and high) scenarios, the coral reef models predicted 2,570–3,377 ha of benthic habitat with decreased coral cover and 64–94.9 t of fish biomass, with an average loss ranging from 3.7–8.8% coral cover and 44.4–54.8 kg.ha^−1^ fish biomass (Table 1). Under combined scenarios, we identified that coral reef areas subject to sediment runoff and bleaching impacts were mostly located around the eastern back-reefs and a nearshore portion of the southern reefs (Fig. 7d and areas with potential cumulative impacts are shown in purple in Fig. 8d). When combining restoration and climate change scenarios (low, moderate, and high), the coral reef models predicted an average loss in coral cover ranging from 3.1–10.2% over 1,351–1,906 ha and an associated fish biomass loss of 13.5–75.5 t (18.4–40.2 kg.ha^−1^) in areas more exposed to climate change impacts (Table 1). Across areas that were not exposed to climate change impacts but were influenced by reduced sediment runoff from restored watersheds, the coral reef models predicted an average increase of 1.7% coral cover over 399–402 ha, with a corresponding increase of 5.3–5.7 t of fish biomass (16.1–19.7 kg.ha^−1^) (Table 1). Under combined scenarios, we identified that coral reef areas benefiting from reduced sediment runoff and subject to bleaching impacts were mostly located around the eastern back-reefs and portions of the fringing reefs (Fig. 7e and area with potential cumulative impacts are shown in purple in Fig. 8e).

### Spatial prioritization of conservation

Under present land-use and all climate change scenarios, 14 to 17 watersheds were influencing the impacted coral reef areas and should be prioritized for conservation (Fig. 8a). Where a significant change was detected on coral reefs under the deforestation scenario, 14 watersheds were identified as drivers of this change, and contained land areas where the change in sediment export was significantly different (Fig. 8b). We identified these areas as priorities for conservation. Where a significant change was detected on coral reefs under the restoration scenario, eight watersheds were identified as drivers of these changes and contained land areas where the sediment export was significantly different (Fig. 8c). We identified these areas as priorities for restoration. Under deforestation and all climate change (low, moderate, and high) scenarios, 18 watersheds were influencing the impacted coral reef areas and several priority land areas were identified for forest conservation (Fig. 8d). We identified these areas as priorities for conservation actions. Under restoration and all climate change (low, moderate, and high) scenarios, 7 watersheds were negatively impacting these coral reef areas and 8 watersheds were positively impacting these coral reef areas, respectively (Fig. 8e). We identified these areas as priorities for conservation and restoration actions.

## Discussion

Integrated land-sea planning requires the ability to trace where land-based pollutants come from and where they are likely to cause an impact once they enter the marine environment. Our modeling framework provides a place-based, high spatial resolution, flexible tool to quantify, track, and map the impact of land-use change on coral reefs at the sub-watershed scale. The field data required to build this model are often collected by monitoring programs of local NGOs or government agencies. While the bathymetry and habitat maps used in this study were derived from purchased remote sensing data and intensively ground-truthed in the field, which makes our models more accurate, these methods can easily be applied with increasingly available free remote sensing imagery and bathymetry data (i.e., Worldview III, GEBCO), making this approach useful for regions with limited funding. In addition, our modeling framework relies on two freely available software packages (i.e., InVEST SDR, and R) and ArcGIS (also available with open access QGIS or GRASS GIS)^59,98,111,120,121^.

By coupling the framework with scenario planning, this tool can be used in partnership with decision-makers to inform local conservation actions and identify priority areas on land that can foster coral reef resilience. The outputs are simple maps and spreadsheets, thereby allowing for more transparency in the decision making process, which can foster community buy-in^50^. For instance, our modeling results identified the Kilaka River watershed as a site where protection of the watershed could have substantial downstream benefits. This watershed was the site of a recent conservation intervention, where a conservation NGO has recently signed a 99-year conservation lease after negotiating with the local landowners^122^. Although the lease was signed mainly to protect to terrestrial biodiversity, the protected area will likely also foster coral reef resilience to climate change and the outputs of these models can be used to communicate these multiple benefits to the communities in the district (see Fig. 7b, northernmost purple watershed), which will provide broader fisheries benefits to Kubulau residents who all have access to the fishing grounds.

The modeling framework calibration results established the effect of current land-use and sediment runoff on coral reefs in the study area. The coral reef models indicated that coralline algae, corals, and turf algae cover, upon which targeted herbivores depend, were negatively related to sediment exposure along with predatory fish biomass (Fig. 6). The adverse direct and indirect impacts of sedimentation and turbidity on benthic habitat at local scales has been well established^11^. Increases in sediment can indirectly hinder competition for space by reef calcifiers^13,123^. Even if coral reefs in turbid waters can flourish^124^, they are restricted to the top 4–10 m depth range^125,126^, and typically support fewer species, slower growth rates, and poorer recruitment^127^. Coral reef fishes can be adversely affected by sedimentation and turbidity through altered foraging^128^. Sedimentation and turbidity also indirectly affect coral reef fishes by altering the benthic community structure and composition^127,129^. The degree of dependence on different benthic groups may influence the susceptibility of fishes to habitat impacts from sediment runoff^40,129,130^ which can decrease or alter fish recruitment^38,131^. Similarly, our coral reef predictions indicated that inshore reefs exposed to high sediment runoff generally support less coral cover and fish biomass, in contrast to offshore reefs which support higher coral cover and fish biomass (Fig. 6)^132^.

It is increasingly recognized that water quality interacts with elevated SST and has a profound influence on management outcomes of nearshore coral reefs under climate change^133^. Thus, informed management requires knowing where the cumulative effect of these human drivers is less than (antagonistic), more than (synergistic), or additive^24,134,135^. This research identified where coral reef areas may be subject to either bleaching, sediment exposure, or both, but did not explicitly model their potential interactions and cumulative impacts due to a lack of nutrient data and the poor understanding of those processes^16–18^. However, it is likely that nutrient enrichment would directly exacerbate bleaching rates through increased disease^136^. In addition, nutrients can contribute to lack of recovery from bleaching through promoting algae growth^14,15^. On the other hand, recent work has shown that sediments can have an antagonistic effect with SST by mitigating bleaching impacts through shading^16,133^. Therefore, the cumulative impacts of sediments and nutrients under elevated SST are challenging to model explicitly. Even so, the absence of change in the inshore areas across scenarios shown in this study suggests that inshore reefs are more resilient to bleaching and sediment runoff. This observed pattern aligns with other empirical research showing that coral reefs chronically exposed to high turbidity are less vulnerable to sediment and bleaching impacts^17,133^.

Identifying where coral reefs are more or less vulnerable to local and global human impacts can inform place-based management actions and spatial prioritization to minimize risks (i.e., probability of disturbance)^137^. For example, a low risk conservation approach would protect the nearshore reefs that are not susceptible to bleaching or sediment impacts^138^, which can act as potential refugia during climate-induced disturbances^17,139^. Many of these inshore reefs are already placed under traditional closures (*tabu*)^62^ (Fig. 1b). A more risky conservation approach would focus on protecting the offshore reefs, which are more vulnerable to bleaching and sediment runoff and prone to interactions^138^, but support more reef calcifiers and fishes (Fig. 6). Most of those coral reef areas also constitute important local fishing grounds for nearby coastal villages^140^ and some have been designated permanent no-take marine closures^62^ (Fig. 1b). To foster the resilience of these reefs, it is essential to consider minimizing land-based impacts, as research increasingly shows that marine closures are less effective when exposed to high land-based source pollution^18,29^. For coral reefs mainly vulnerable to climate change impacts, adopting marine conservation actions that protect key ecosystem processes and functions, like grazing from herbivores, can foster recovery post-bleaching events^65,139,141^. As highlighted by Game *et al*.^137^, conservation planning should design protected areas that go beyond protecting parts of the ecosystem within their boundaries that are resilient to climate change and instead augment resilience on a scale that transcends land-sea boundaries.

Gaining knowledge of where soils are more likely to erode under land-use change can inform where conservation actions on land or sustainable land-use practices can provide benefits downstream. The scenario analysis results identified priority land areas within 14 priority watersheds (out of 26 modeled watersheds) where forest conservation and or restoration can foster resilience of coral reefs vulnerable to projected bleaching impacts (Fig. 8). Conserving forest in priority land areas of these key watersheds not only maintains good water quality essential for healthy coral reefs, it is also an important management strategy to mitigate the impacts of climate change on nearshore coral reefs within the range of sediment dispersal from river plumes^123,142^. Although our scenarios focused on Kubulau District, three large watersheds where sediment discharge from mining activity has impacted coral reefs in Kubulau through drainage from the Yanawai River are located outside the governance of Kubulau District (Fig. 1). This mismatch of governance and natural boundaries/processes can result in decision-makers having no control over activities outside their jurisdiction that impact the ecology of their systems^143^.

Uncertainty is inherent in modeling complex systems^144^ and arises at all stages of the modeling process^145^. A predictive model calibrated on present conditions can be used to forecast potential species distribution at another point in time^104^, but requires a number of assumptions given its static nature. One of the foremost assumptions associated with predicting futures with static models is that species distributions are in equilibrium with current conditions and the identified relationships will not change over time^104,146^, which may not always be true^147^. For example, evidence is emerging that corals may acclimatize to predicted increases in SST associated with climate change^77^, in which case, these results may overestimate impact and the recommended management interventions may not be as applicable. Nevertheless, our bleaching scenarios were conservative compared to projected coral bleaching impacts in Hawaiʻi and historical data from Fiji. In addition, bleaching impact was uniformly implemented across specific depth-ranges, when research shows that coral-bleaching impacts are expected to worsen heterogeneously in the future given increases in SST^1,148^. Although our sediment models accounted for the connectivity across the landscape, our coral reef spatial predictive modeling did not account for exchange between grid-cells, such as species dispersal, migration, and interactions within the seascape^34,149^. Meaning, our framework does not provide information on recovery trajectories of impacted ecosystems^48,145^.

Another key assumption associated with predicting futures with static models is that the effects of time lags and the complex, nonlinear relationships between land-use practices and coral reef benefits are not accounted for, which can influence management scale and outcomes^150^. For instance, the deforestation and restoration scenarios were extreme and assumed all clearing and restoration was immediate. In reality, clearing proceeds in a patchwork over time and different areas would have different amounts of ground cover or regrowth at different times^56^, therefore sediment export would differ from the total export modeled here. Similarly, restoration is not an instantaneous process, as it will take many years before a forest has established enough to provide the sediment retention and runoff reduction we considered. From a marine perspective, coral reef response to change in sediment runoff will also vary over time based on taxon physiology and environmental conditions^16^. Thus, from a management perspective, it is essential to account for the timeframe of anticipated outcomes of conservation actions to factor in social and economic constraints^54^. Our findings indicate that forest conservation actions on land should be a high priority because they promote coral reef resilience^151^. However, we only explicitly considered land-use change in terms of local actions in our scenarios and did not evaluate the potential benefits from marine-based conservation actions.

Given imperfect knowledge of both the effect of land-based pollution and climate change on coral reefs^152^, scenario modeling requires simplifications and assumptions which lead to uncertainty in model projections. Although we used present conditions as the baseline for examining projected coral reefs, this comparative benchmark represents potentially impacted ecosystems^153^. However, present conditions still provide an opportunity to identify the trajectory of coral reefs under different human drivers and provide guidance for management^154^. Given that sources of uncertainty in scenario analysis are inevitable, we used scenario modeling to illustrate the range of possibilities for the future of coral reefs. This approach spatially identified the drivers of coral reef degradation and provided guidance on priority locations where local management could be most effective. Additionally, these findings can help achieve more effective management outcomes by indicating where coral reefs and associated marine resources may be at higher risk^145,152^.

This modeling framework was developed under several key data gaps and caveats. First, no *in situ* water quality data was available for the streams and coastal waters modeled, which prevented us from ground-truthing the sediment and coastal plume models. The sediment loadings for each watershed may have under-estimated some sediment export loads due to erosion processes that we did not account for in our modeling approach, such as land slip and stream bank erosion which can be major sources of sediment^42,155^. Landslides additionally represent a potentially large and unexplored source of fine sediment in Fiji. Approximately 67% of Viti Levu is reported to be above 18 degrees slope, which some authors (e.g. Morrison *et al*.^156^) use as a threshold for instability. Deforestation on steep slopes has the potential to catastrophically destabilize large areas because of the role that roots can play in binding shallow (e.g. 2–3 m) subsurface soils^157^. Similarly, no data on coastal oceanographic processes were available for Kubulau at a fine spatial resolution. The coastal plume models could over- or under-estimate the TSS proxy values because we could not account for the effect of longshore and tidal-driven transport on sediment dispersal and settling rates due to lack of data. To overcome some of those limitations, we used proxies of sediment dispersal such as depth, distance from river mouth, and exposure to local predominant wave and wind regimes. Our comparisons of the coral reef indicators observations and predictions accounted for between 23–51% of the total variation and were all statistically significant (Figs 6 and S9). The relative consistency in the coral reef spatial predictions, compared to the observed indicator values, provided some confidence in the relative change in distribution under different scenarios. The modeling outputs can be easily understood by local communities and their feedback can be used for further ground-truthing these patterns. Future work should investigate how these modeled plumes of TSS compare to local knowledge from coastal communities, satellite imagery, and/or *in situ* data as those become available.

We also tested how sensitive our modeling framework was to the linkages between the SDR model and the coral reef models by running the framework with various cfactor values for the key land-uses that change across scenarios (i.e., native forest, pine plantations, monoculture of taro and kava, and shrubland). We observed that the magnitude of change and the sizes of the spatial footprints of coral reef impacts as a function of sediment runoff varied depending on the cfactors used. However, the directionality of change in the coral reef indicators was consistent. Similarly, the locations of coral reef impacts from sediment runoff were consistently detected near the watersheds that contributed the largest change in sediment export. Therefore, the priority areas on land within watersheds linked to vulnerable coral reef areas did not change. It has been established in environmental decision theory that uncertainties in the input parameters can alter predictions but do not change the relative priority of management options^54,158,159^. Our approach is sensitive to small changes, because we used species distribution models that can describe ecological relationships at a fine spatial scale and the scenario impact analysis relies on relative differences^104,117^. Based on these trends, we concluded that this framework can reliably identify priority areas to undertake further field investigations and inform place-based conservation actions.

## Conclusion

There is a growing need to develop planning tools to help prioritize local conservation actions at relevant spatial scales for decision makers. This approach offers a data-driven, place-based model that is spatially-explicit and can easily be updated as more data become available. Although static in nature, this approach can complement and at times offer several advantages over simple GIS-based models^41^ and more complex dynamic biophysical models^34,42^. The coral reef models represent the ecological relationships specific to a place that can then be used to calibrate dynamic biophysical models. Another key strength of this approach is that it estimates the impact of land-use change on coral reef ecosystems at fine spatial resolution (60 × 60 m). By simultaneously evaluating the effect of land-use change, sediment runoff, coral reef habitat, and associated fish communities, this research highlighted the potential trade-offs and synergies arising between land and sea under different land-use scenarios. The next steps would be to build a suite of land-use management scenarios within the priority areas we identified in this study. Then, evaluate tradeoffs to identify optimal management solutions. By adopting a ridge-to-reef conservation planning process, protected areas can be designed for multiple benefits that include improvements in biodiversity, drinking water, and reef fisheries^60,71^.

Our findings support the paradigm that local, place-based management can promote coral reef resilience to climate change^2,160^. These findings can also help inform priorities for future conservation leases or other payment for ecosystem service schemes by^55^: (1) identifying relevant communities, (2) facilitating communication using maps as visuals^161^, and (3) locating where forest conservation or restoration actions can benefit coral reefs and improve fisheries livelihoods^162^. The implementation of this approach in GIS allows managers to visualize and foresee the potential outcomes of management interventions. This type of approach has the potential to engender collaborative stewardship among agencies, communities, and NGOs. Due to limited data on the influence of land-use change on coastal water quality to support land-sea integrated planning in Fiji and limited funding for further data collection, the ICM process in Bua Province will benefit from this assessment of where land-use change may have the greatest impact on coral reefs in Kubulau District. While the case study is spatially limited, this framework is easily transferable to other oceanic island systems. The framework presented here is a promising a decision support tool in the context of land-sea integrated management of coral reef ecosystems.

## Electronic supplementary material

Supplementary Information
