# Supplementary material for: Scenario planning with linked land-sea models inform where forest conservation actions will promote coral reef resilience
Source: Sci Rep. 2018 Aug 20;8:12465. doi: 10.1038/s41598-018-29951-0 (PMC6102229; doi:10.1038/s41598-018-29951-0)
Supplement: Supplementary file 1 — Supplementary Information [file 41598_2018_29951_MOESM1_ESM.docx]

**Scenario planning with linked land-sea models inform where forest conservation actions will promote coral reef resilience**

Delevaux J.M.S. ^1,2^*, Jupiter S. D.^3^, Stamoulis K.A.^4,5^, Bremer L.L.^6,7^, Wenger A.S.^8^, Dacks R.^9^, Garrod P. ^10^, Falinski K.A.^11^, Ticktin T.^1^

^1^Department of Botany, University of Hawaiʻi, Honolulu, Hawaiʻi, USA

^2^School of Ocean and Earth Science and Technology, University of Hawaiʻi, Honolulu, Hawaiʻi, USA

^3^Wildlife Conservation Society, Melanesia Program, 11 Ma’afu Street, Suva, Fiji

^4^School of Molecular and Life Sciences, Curtin University, Perth, Australia

^5^Fisheries Ecology Research Lab, University of Hawaiʻi, Honolulu, Hawaiʻi, USA

^6^University of Hawaiʻi Economic Research Organization, University of Hawaiʻi, Honolulu, Hawaiʻi, USA

^7^University of Hawaiʻi Water Resources Research Center, University of Hawaiʻi, Honolulu, Hawaiʻi, USA

^8^School of Earth and Environmental Sciences, University of Queensland, Brisbane, QLD, Australia

^9^Department of Biology, University of Hawaiʻi, Honolulu, Hawaiʻi, USA

^10^Department of Natural Resources and Environmental Management, University of Hawaiʻi, Honolulu, Hawaiʻi, USA

^11^The Nature Conservancy, Hawaii Marine Program, Honolulu, Hawaiʻi, USA

* Corresponding author

E-mail: [jademd@hawaii.edu](mailto:jademd@hawaii.edu); +1-808-372-5794

# Supplementary Information overview:

Supplementary Methods – Coral reef field data; Fig S1. Response curves for coralline algae; Fig S2. Response curves for coral; Fig S3. Response curves for macroalgae; Fig S4. Response curves for turf algae; Fig S5. Response curves for browsers; Supplementary Fig S6. Response curves for grazers; Fig S7. Response curves for scrapers; Fig S8. Response curves for predators; Fig S9. Coral reef prediction validation; Table S1. Coral reef benthic and fish indicators coupled with descriptions on how they are affected by sediment and/or confer resilience to sediment or other impacts; Table S2. Fish species composition per functional groups; Table S3. C-factors used in InVEST Sediment Delivery Ratio model and their respective sources; Table S4. Description of marine drivers; Table S5. Coral reef models calibration and cross-validation (CV) percent deviance explained (PDE).

## Supplementary Methods – Coral reef field data

Generally, two forereef and two backreef sites were surveyed within each management treatment, with 3-5 replicate transects nested within depth categories and depth nested within sites. At each site, 50 x 5 m belt transects (250-m^2^ transect area) were used to collect benthic and fish data. Benthic life-form categories were recorded along the 50 m transects at 0.5 m intervals ^1^. Life-form classes were reclassified according to the following 8 functional strata: crustose coralline algae (CCA, including coralline algae with structure); live hard scleractinian coral (COR: *Acropora* spp., branching, corymbose, encrusting, foliose, massive, and submassive corals); macroalgae (MAC: all fleshy macroalgae *>*2 cm, dead coral with fleshy algae); turf algae (TUR: *≤*2 cm height on dead coral or reef pavement); filter feeders (FLF: sponge, soft corals, zooanthids), hard bottom (HB: rubble, non-carbonate rock); soft bottom (SB: sand, silt); and other substrate (OT: including *Halimeda* spp., microbial and other biota). For each belt transect, divers recorded total length (TL) of observed fishes for each targeted species, using 5 cm length classes for fishes < 40 cm and exact size ≥40 cm^2^. To calculate the biomass for each fish, we applied length estimates in the length-weight (L-W) expression $W = a \times L^{b}$, where a and b are constants for the allometric growth equation, L is total length in cm, and W is mass in kg, using species-specific *a* and *b* parameters obtained from FishBase^3^ with *a* and *b* parameter values preferentially selected from sites closest to Fiji (e.g., New Caledonia)^4^. As many of the L–W conversions required fork length (FL), a length–length (L–L) conversion factor was obtained from FishBase where necessary to convert from total length (TL) recorded during the surveys to FL before biomass estimation {Citation}. Because the biomass conversion formula resulted in some grossly overestimated weights for fishes that substantially change morphology as they age, maximum published weights were used for certain species when these fish were sighted above threshold sizes, as per Jupiter and Egli (2011)^2^.

# Supplementary Fig S1. Response curves for coralline algae. Curves show the relative relationships with selected terrestrial and marine drivers.

# Supplementary Fig S2. Response curves for coral. Curves show the relative relationships with selected terrestrial and marine drivers.

# Supplementary Fig S3. Response curves for macroalgae. Curves show the relative relationships with selected terrestrial and marine drivers.

# Supplementary Fig S4. Response curves for turf algae. Curves show the relative relationships with selected terrestrial and marine drivers.

# Supplementary Fig S5. Response curves for browsers. Curves show the relative relationships with selected terrestrial and marine drivers.

# Supplementary Fig S6. Response curves for grazers. Curves show the relative relationships with selected terrestrial and marine drivers.

# Supplementary Fig S7. Response curves for scrapers. Curves show the relative relationships with selected terrestrial and marine drivers.

# Supplementary Fig S8. Response curves for predators. Curves show the relative relationships with selected terrestrial and marine drivers.

# Supplementary Fig S9. Coral reef prediction validation

# Supplementary Table S1. Coral reef benthic and fish indicators coupled with descriptions on how they are affected by sediment and/or confer resilience to sediment or other impacts.

| Type | Code | Metric | Ecological value | Reference |
| --- | --- | --- | --- | --- |
| Benthic indicators | CCA | Coralline algae | CCA and corals are active reef builders, which can foster recovery, larvae recruitment, and provide habitat for reef fishes. Corals are sensitive to sediment, Reduced water clarity can reduce their depth range. Sediment can smoother corals. | ^6–8^ |
|  | COR | Coral cover |  |  |
|  | MAC | Macroalgae | Macroalgae can benefit from nutrient binding with sediment and interact with corals through allelopathy, competition with coral reduces space for recruits, and chemical cues that reduce coral and fish settlement. Turf is important food source for herbivores and may be smothered by sedimentation. | ^8–13^ |
|  | TUR | Turf algae |  |  |
| Fish indicators | BROW | Browsers | Surgeonfishes and most parrotfishes graze on turf algae or macroalgae, which can help reverse coral-algal phase shifts and some parrotfishes free space for CCA and coral larval settlement. | ^5^ |
|  | GRDT | Grazers |  |  |
|  | SCEX | Scrapers |  |  |
|  | PISC | Predator | High biomass of predator can be a sign of a healthy and not overfished population | ^20^ |

Benthic (% cover) and fish biomass (kg.ha^-1^) coral reef indicators were derived from the coral reef surveys and used as response variables in the coral reef models.

# Supplementary Table S2. Fish species composition per functional groups

| **Function** | **Family** | **Scientific name** | **Common name** |
| --- | --- | --- | --- |
| **Browsers** | Acanthuridae | *Naso annulatus* | Whitemargin unicornfish |
|  |  | *Naso brachycentron* | Humpback unicornfish |
|  |  | *Naso lituratus* | Orangespine unicornfish |
|  |  | *Naso sp.* | Unicornfish spp. |
|  |  | *Naso tonganus* | Bulbnose unicornfish |
|  |  | *Naso tuberosus* | Humpnose unicornfish |
|  |  | *Naso unicornis* | Bluespine unicornfish |
|  | Kyphosidae | *Kyphosus bigibbus* | Brown chub |
|  |  | *Kyphosus cinerascens* | Blue sea chub |
|  |  | *Kyphosus sp.* | Chubs spp. |
|  |  | *Kyphosus vaigiensis* | Brassy chub |
| **Grazers** | Acanthuridae | *Acanthurus auranticavus* | Orange-socket surgeonfish |
|  |  | *Acanthurus blochii* | Ringtail surgeonfish |
|  |  | *Acanthurus fowleri* | Fowler's surgeonfish |
|  |  | *Acanthurus grammoptilus* | Finelined surgeonfish |
|  |  | *Acanthurus leucocheilus* | Palelipped surgeonfish |
|  |  | *Acanthurus leucopareius* | Whitebar surgeonfish |
|  |  | *Acanthurus lineatus* | Lined surgeonfish |
|  |  | *Acanthurus maculiceps* | White-freckled surgeonfish |
|  |  | *Acanthurus nigricans* | Goldrim surgeonfish |
|  |  | *Acanthurus nigricauda* | Epaulette surgeonfish |
|  |  | *Acanthurus nigrofuscus* | Brown surgeonfish |
|  |  | *Acanthurus nigroris* | Bluelined surgeonfish |
|  |  | *Acanthurus olivaceus* | Orangeband surgeonfish |
|  |  | *Acanthurus pyroferus* | Chocolate surgeonfish |
|  |  | *Acanthurus sp.* | Surgeonfish spp. |
|  |  | *Acanthurus triostegus* | Convict surgeonfish |
|  |  | *Acanthurus xanthopterus* | Yellowfin surgeonfish |
|  |  | *Zebrasoma flavescens* | Yellow tang |
|  |  | *Zebrasoma scopas* | Twotone tang |
|  |  | *Zebrasoma sp.* | Tang spp. |
|  |  | *Zebrasoma veliferum* | Sailfin tang |
|  | Siganidae | *Siganus argenteus* | Rabbitfish |
|  |  | *Siganus doliatus* | Barred spinefoot |
|  |  | *Siganus guttatus* | Orange-spotted spinefoot |
|  |  | *Siganus punctatissimus* | Peppered spinefoot |
|  |  | *Siganus punctatus* | Goldspotted spinefoot |
|  |  | *Siganus sp.* | Spinefoot spp. |
|  |  | *Siganus spinus* | Little spinefoot |
|  |  | *Siganus stellatus* | Brown-spotted spinefoot |
|  |  | *Siganus uspi* | Bicolored foxface |
|  |  | *Siganus vermiculatus* | Vermiculated spinefoot |
| **Scrapers** | Scaridae | *Cetoscarus bicolor* | Bicolour parrotfish |
|  |  | *Chlorurus bleekeri* | Bleeker's parrotfish |
|  |  | *Chlorurus frontalis* | Pacific slopehead parrotfish |
|  |  | *Chlorurus japanensis* | Palecheek parrotfish |
|  |  | *Chlorurus microrhinos* | Steephead parrots |
|  |  | *Chlorurus sordidus* | Pacific bullethead parrotfish |
|  |  | *Chlorurus sp.* | Parrotfish spp. |
|  |  | *Hipposcarus longiceps* | Pacific longnose parrotfish |
|  |  | *Hipposcarus sp.* |  |
|  |  | *Scarus altipinnis* | Filament-finned parrotfish |
|  |  | *Scarus chameleon* | Chameleon parrotfish |
|  |  | *Scarus dimidiatus* | Yellowbarred parrotfish |
|  |  | *Scarus forsteni* | Forsten's parrotfish |
|  |  | *Scarus frenatus* | Bridled parrotfish |
|  |  | *Scarus ghobban* | Blue-barred parrotfish |
|  |  | *Scarus globiceps* | Globehead parrotfish |
|  |  | *Scarus guttatus* | Blue-barred parrotfish |
|  |  | *Scarus japanensis* | Palecheek parrotfish |
|  |  | *Scarus longipinnis* | Highfin parrotfish |
|  |  | *Scarus niger* | Dusky parrotfish |
|  |  | *Scarus oviceps* | Dark capped parrotfish |
|  |  | *Scarus prasiognathos* | Singapore parrotfish |
|  |  | *Scarus psittacus* | Palenose parrotfish |
|  |  | *Scarus rivulatus* | Midnight parrotfish |
|  |  | *Scarus rubroviolaceus* | Ember parrotfish |
|  |  | *Scarus schlegeli* | Yellowband parrotfish |
|  |  | *Scarus sp.* | Parrotfish spp. |
|  |  | *Scarus spinus* | Greensnout parrotfish |
| **Predator** | Carangidae | *Alectis ciliaris* | Threadfin trevally |
|  |  | *Carangoides ferdau* | Blue trevally |
|  |  | *Carangoides fulvoguttatus* | Yellowspotted trevally |
|  |  | *Carangoides gymnostethus* | Bludger |
|  |  | *Carangoides oblongus* | Coachwhip trevally |
|  |  | *Carangoides plagiotaenia* | Barcheek trevally |
|  |  | *Caranx ignobilis* | Giant trevally |
|  |  | *Caranx melampygus* | Bluefin trevally |
|  |  | *Caranx papuensis* | Brassy trevally |
|  |  | *Caranx sexfasciatus* | Bigeye trevally |
|  |  | *Caranx sp.* | Trevally spp. |
|  |  | *Elagatis bipinnulata* | Rainbow runner |
|  |  | *Gnathanodon speciosus* | Golden trevally |
|  |  | *Scomberoides lysan* | Doublespotted queenfish |
|  |  | *Seriola rivoliana* | Almaco jack |
|  |  | *Trachinotus baillonii* | Smallspotted dart |
|  |  | *Trachinotus blochii* | Snubnose pompano |
|  |  | *Uraspis helvola* | Whitetongue jack |
|  | Carcharhinidae | *Carcharhinus amblyrhynchos* | Blacktail reef shark |
|  | Labridae | *Epibulus insidiator* | Sling-jaw wrasse |
|  |  | *Oxycheilinus bimaculatus* | Two-spot wrasse |
|  |  | *Oxycheilinus digrammus* | Cheeklined wrasse |
|  |  | *Oxycheilinus orientalis* | Oriental maori wrasse |
|  |  | *Oxycheilinus sp.* | Wrasse spp. |
|  | Lethrinidae | *Lethrinus atkinsoni* | Pacific yellowtail emperor |
|  |  | *Lethrinus erythracanthus* | Orange-spotted emperor |
|  |  | *Lethrinus erythropterus* | Longfin emperor |
|  |  | *Lethrinus harak* | Thumbprint emperor |
|  |  | *Lethrinus laticaudis* | Grass emperor |
|  |  | *Lethrinus lentjan* | Pink ear emperor |
|  |  | *Lethrinus microdon* | Smalltooth emperor |
|  |  | *Lethrinus miniatus* | Trumpet emperor |
|  |  | *Lethrinus nebulosus* | Spangled emperor |
|  |  | *Lethrinus obsoletus* | Orange-striped emperor |
|  |  | *Lethrinus olivaceus* | Longface emperor |
|  |  | *Lethrinus semicinctus* | Black blotch emperor |
|  |  | *Lethrinus sp.* | Emperor spp. |
|  |  | *Lethrinus xanthochilus* | Yellowlip emperor |
|  | Lutjanidae | *Aphareus furca* | Small-toothed jobfish |
|  |  | *Aprion virescens* | Green jobfish |
|  |  | *Lutjanus argentimaculatus* | Mangrove red snapper |
|  |  | *Lutjanus biguttatus* | Two-spot banded snapper |
|  |  | *Lutjanus bohar* | Two-spot red snapper |
|  |  | *Lutjanus ehrenbergii* | Blackspot snapper |
|  |  | *Lutjanus fulviflamma* | Dory snapper |
|  |  | *Lutjanus fulvus* | Blacktail snapper |
|  |  | *Lutjanus gibbus* | Humpback red snapper |
|  |  | *Lutjanus johnii* | John's snapper |
|  |  | *Lutjanus kasmira* | Common bluestripe snapper |
|  |  | *Lutjanus monostigma* | One-spot snapper |
|  |  | *Lutjanus quinquelineatus* | Five-lined snapper |
|  |  | *Lutjanus rivulatus* | Blubberlip snapper |
|  |  | *Lutjanus russeli* | Russell's snapper |
|  |  | *Lutjanus semicinctus* | Black-banded snapper |
|  |  | *Lutjanus sp.* | Naso |
|  | Mullidae | *Mulloidichthys flavolineatus* | Yellowstripe goatfish |
|  |  | *Mulloidichthys sp.* | Goatfish spp. |
|  |  | *Mulloidichthys vanicolensis* | Yellowfin goatfish |
|  |  | *Parupeneus cyclostomus* | Blue goatfish |
|  | Serranidae | *Anyperodon leucogrammicus* | Slender grouper |
|  |  | *Belonoperca chabanaudi* | Arrowhead soapfish |
|  |  | *Cephalopholis argus* | Blue spotted grouper |
|  |  | *Cephalopholis leopardus* | Leopard hind |
|  |  | *Cephalopholis miniata* | Coral trout |
|  |  | *Cephalopholis sexmaculata* | Sixblotch hind |
|  |  | *Cephalopholis sp.* | Grouper spp. |
|  |  | *Cephalopholis urodeta* | Darkfin hind |
|  |  | *Gracila albomarginata* | Masked Grouper |
|  |  | *Plectropomus areolatus* | Squaretail coralgrouper |
|  |  | *Plectropomus laevis* | Blacksaddled coralgrouper |
|  |  | *Plectropomus leopardus* | Leopard coralgrouper |
|  |  | *Plectropomus maculatus* | Spotted coralgrouper |
|  |  | *Plectropomus pessuliferus* | Roving coralgrouper |
|  |  | *Variola albimarginata* | White-edged lyretail |
|  |  | *Variola louti* | Yellow-edged lyretail |
|  | Sphyraenidae | *Sphyraena barracuda* | Great barracuda |
|  |  | *Sphyraena flavicauda* | Yellowtail barracuda |
|  |  | *Sphyraena qenie* | Blackfin barracuda |
|  |  | *Sphyraena sp.* | Barracuda spp. |

# Supplementary Table S3. C-factors used in InVEST Sediment Delivery Ratio model and their respective sources.

| **Land Cover type** | **C factor** | **P factor** | **Citation** |
| --- | --- | --- | --- |
| Coconuts | 0.2 | 1 | FAO 2000 |
| Fallow | 0.12 | 1 | ^14^; Natural Resource Conservation Service |
| Fish pond | 0.001 | 1 | ^15^ |
| Natural Forest | 0.002 | 1 | ^16^; ^17^ |
| Secondary forest | 0.0045 | 1 | "Bosque secundario"; avg of listed values ^17^; FAO 2000 |
| Pine | 0.007 | 1 | ^14,17,18^ |
| Hardwood Timber Plantation | 0.03 | 1 | "Bosque degradado"; avg of listed values ^17^ |
| Polyculture | 0.3 | 1 | ^16^ |
| Monoculture taro | 0.4 | 1 | used cassava values from ^16^ and potato value from ^17^ |
| Monoculture kava | 0.4 | 1 | used cassava values from ^16^ and potato value from ^17^ |
| Monoculture Rice | 0.2 | 1 | Adopting rice standards ^17^ |
| Grassland | 0.012 | 1 | ^17^ |
| shrubland | 0.008 | 1 | ^18^ |
| Village | 0.2 | 1 | ^16^ |
| Mangroves | 0.002 | 1 | used the same c-factor as the natural forest value |
| Wetland | 0.001 | 1 | ^19^ |

# Supplementary Table S4. Description of marine drivers. Each metric is classified by type (terrestrial or marine driver). Each metric was coded for modeling. The table below indicates the data source and analytical tool used to generate each metric. Refer to Stamoulis & Delevaux et al. *in press* for more details on processing methods.

| **Type** | **Drivers^a^** | **Description** | **Analytical tool** | **Unit** | **Code** |
| --- | --- | --- | --- | --- | --- |
| **Geography** | Depth^b^ | Mean seafloor depth | ArcGIS Spatial Analyst tools ^20^ | m | Depth |
|  | Distance to shore^c^ | Euclidean distance to the shoreline | ArcGIS Spatial Analyst Euclidean Distance tool ^20^ | m | dist2shore |
| **Habitat topography** | BPI^b^ | Relative topographic position of a point based its elevation and the mean elevation within a neighborhood (m) | Benthic Terrain Modeler tool ^21^ | m | bpi |
|  | Slope^b^ | Maximum rate of change in seafloor depth between each grid cell and its neighbors | ArcGIS Slope tool ^20^  ArcGIS Focal Statistics tool ^20^ | Degrees | slp |
| **Habitat complexity** | Slope of slope^b^ | Second derivative of slope. | ArcGIS Spatial Analyst tools (ESRI 2011) | Degrees | slpslp |
|  | Planar curvature^b^ | Seafloor curvature perpendicular to the direction of the maximum slope (mean). Value indicates whether flow will converge or diverge over a point. | DEM Surface Tools Curvature tool ^22^ | Radians.m^-1^ | curv_plan |
|  | Profile curvature^b^ | Seafloor curvature in the direction of the maximum slope (mean). Value indicates whether flow will accelerate or decelerate over the curve. |  | Radians.m^-1^ | curv_pro |
| **Habitat exposure** | Aspect^b^ | Downslope direction of maximum rate of change in seafloor depth between each grid cell and its neighbors (sine circular mean, cosine circular mean, circular standard deviation) | ArcGIS Aspect tool ^20^; ArcGIS Spatial Analyst tools (sine and cosine function) ^20^ | Degrees | asp_sin, asp_cos, asp_sd |
| **Habitat connectivity** | Contiguity^d^ | Mean spatial connectedness of patches based on the spatial connectedness of cells within a patch; large contiguous patches will result in larger contiguity index values | Exhaustive sampling using a 60m radius moving window analysis in Fragstats v4.2 | Unitless | contig_mn |
|  | Fractal dimension^d^ | Mean patch complexity at the landscape level |  |  | frac_mn |
|  | Proximity^d^ | Measure of patch isolation |  |  | prox_mn |
|  | Shannon diversity index^d^ | Diversity of benthic cover types in the landscape |  |  | shdi |

^a^ The marine drivers were generated at 60x60 m resolution

^b^ Bathymetry at 4 m native resolution ^23^

^c^ Coastline ^24^

^d^ Habitat composition ^25^

# Supplementary Table S5. Coral reef models calibration and cross-validation (CV) percent deviance explained (PDE). The final number of predictors (Xi) is also indicated.

| **Reef indicators** | **PDE (%)** | **CV PDE (%)** | **Xi** |
| --- | --- | --- | --- |
| CCA | 73.3 | 42.8 | 6 |
| Corals | 81.5 | 50.5 | 4 |
| Macroalgae | 73.1 | 23.6 | 4 |
| Turf algae | 58.5 | 12.9 | 4 |
| Browser | 54.9 | 13.6 | 5 |
| Grazer | 62.4 | 28.3 | 6 |
| Scraper | 29.7 | 12.0 | 8 |
| Predator | 61.0 | 31.7 | 4 |

References

1. English, S. S., Wilkinson, C. C., Baker, V. V. & others. *Survey manual for tropical marine resources*. (Australian Institute of Marine Science (AIMS), 1994).

2. Jupiter, S. D. & Egli, D. P. Ecosystem-based management in Fiji: successes and challenges after five years of implementation. *J. Mar. Biol.* **2011,** (2011).

3. Froese, R. & Pauly, D. *FishBase*. (2011).

4. Jupiter, S. D., Weeks, R., Jenkins, A. P., Egli, D. P. & Cakacaka, A. Effects of a single intensive harvest event on fish populations inside a customary marine closure. *Coral Reefs* **31,** 321–334 (2012).

5. Green, A. L. & Bellwood, D. R. *Monitoring functional groups of herbivorous reef fishes as indicators of coral reef resilience : a practical guide for coral reef managers in the Asia Pacific region*. (IUCN, 2009).

6. Acevedo, R., Morelock, J. & Olivieri, R. A. Modification of Coral Reef Zonation by Terrigenous Sediment Stress. *PALAIOS* **4,** 92–100 (1989).

7. Smith, J. E. *et al.* Re-evaluating the health of coral reef communities: baselines and evidence for human impacts across the central Pacific. *Proc R Soc B* **283,** 20151985 (2016).

8. Fabricius, K. E. Effects of terrestrial runoff on the ecology of corals and coral reefs: review and synthesis. *Mar. Pollut. Bull.* **50,** 125–146 (2005).

9. Rasher, D. B., Stout, E. P., Engel, S., Kubanek, J. & Hay, M. E. Macroalgal terpenes function as allelopathic agents against reef corals. *Proc. Natl. Acad. Sci.* **108,** 17726–17731 (2011).

10. Bonaldo, R. M. & Hay, M. E. Seaweed-coral interactions: variance in seaweed allelopathy, coral susceptibility, and potential effects on coral resilience. *PLoS One* **9,** e85786 (2014).

11. Dixson, D. L., Abrego, D. & Hay, M. E. Chemically mediated behavior of recruiting corals and fishes: a tipping point that may limit reef recovery. *Science* **345,** 892–897 (2014).

12. Birrell, C. L., McCook, L. J., Willis, B. L. & Diaz-Pulido, G. A. Effects of benthic algae on the replenishment of corals and the implications for the resilience of coral reefs. *Ocean. Mar Biol Annu Rev* **46,** 25–63 (2008).

13. Vermeij, M. J. A., Smith, J. E., Smith, C. M., Thurber, R. V. & Sandin, S. A. Survival and settlement success of coral planulae: independent and synergistic effects of macroalgae and microbes. *Oecologia* **159,** 325–336 (2009).

14. Roose, E. J. Application of the universal soil loss equation of Wischmeier and Smith in West Africa. in *Soil Conservation and Management in the Humid Tropics; Proceedings of the International Conference* (1977).

15. Falinski, K. Predicting sediment export into tropical coastal ecosystems to support ridge-to-reef management. (University of Hawaii at Manoa, 2016).

16. FAO. Strategic environmental assessment. (2007). Available at: http://www.fao.org/docrep/007/y2413e/y2413e09.htm. (Accessed: 4th December 2017)

17. Lianes, E., Marchamalo, M. & Roldán, M. Evaluación del factor C de la RUSLE para el manejo de coberturas vegetales en el control de la erosión en la cuenca del río Birrís, Costa Rica. *Agron. Costarric.* **33,** (2009).

18. Chicas, S. & Omine, K. Forest Cover Change and Soil Erosion in Toledo’s Rio Grande Watershed. *Int. Arch. Photogramm. Remote Sens. Spat. Inf. Sci.* **40,** 353 (2015).

19. Wischmeier, W. H. & Smith, D. D. Predicting rainfall erosion losses-a guide to conservation planning. *Predict. Rainfall Eros. Losses- Guide Conserv. Plan.* (1978).

20. ESRI. *ArcGIS Desktop: Release 10. Environmental Systems Research Institute.* (2011).

21. Wright, D. J. *et al.* ArcGIS Benthic Terrain Modeler [a collection of tools used with bathymetric data sets to examine the deepwater benthic environment]. *Or. State Univ. Davey Jones’ Locker Seafloor MappingMarine GIS Lab. NOAA Coast. Serv. Cent.* (2005).

22. Jenness, J. DEM surface tools for ArcGIS. *Jenness Enterp.* 1–96 (2013).

23. Roelfsema, C., Phinn, S., Jupiter, S., Comley, J. & Albert, S. Mapping coral reefs at reef to reef-system scales, 10s–1000s km2, using object-based image analysis. *Int. J. Remote Sens.* **34,** 6367–6388 (2013).

24. Fiji Lands Department. Coastline.

25. Knudby, A., Roelfsema, C., Lyons, M., Phinn, S. & Jupiter, S. Mapping fish community variables by integrating field and satellite data, object-based image analysis and modeling in a traditional Fijian fisheries management area. *Remote Sens.* **3,** 460–483 (2011).
